# Palladium(II) Catalyzed Cyclization-Carbonylation-Cyclization Coupling Reaction of (*ortho*-Alkynyl Phenyl) (Methoxymethyl) Sulfides Using Molecular Oxygen as the Terminal Oxidant

**DOI:** 10.3390/molecules21091177

**Published:** 2016-09-05

**Authors:** Rong Shen, Taichi Kusakabe, Tomofumi Yatsu, Yuichiro Kanno, Keisuke Takahashi, Kiyomitsu Nemoto, Keisuke Kato

**Affiliations:** Faculty of Pharmaceutical Sciences, Toho University, 2-2-1 Miyama, Funabashi, Chiba 274-8510, Japan; shenrong_927@yahoo.co.jp (R.S.); taichi.kusakabe@phar.toho-u.ac.jp (T.K.); 3014004y@nc.toho-u.ac.jp (T.Y.); ykanno@phar.toho-u.ac.jp (Y.K.); keisuke.takahashi@phar.toho-u.ac.jp (K.T.); kiyomitsu.nemoto@phar.toho-u.ac.jp (K.N.)

**Keywords:** palladium, carbonylation, molecular oxygen, CCC-coupling reaction, bisoxazoline

## Abstract

An efficient Pd^II^/Pd^0^-*p*-benzoquinone/hydroquinone-CuCl_2_/CuCl catalyst system was developed that uses environmentally friendly molecular oxygen as the terminal oxidant to catalyze the cyclization-carbonylation-cyclization coupling reaction (CCC-coupling reaction) of (*o*-alkynyl phenyl) (methoxymethyl) sulfides.

## 1. Introduction

**Scheme 1:**
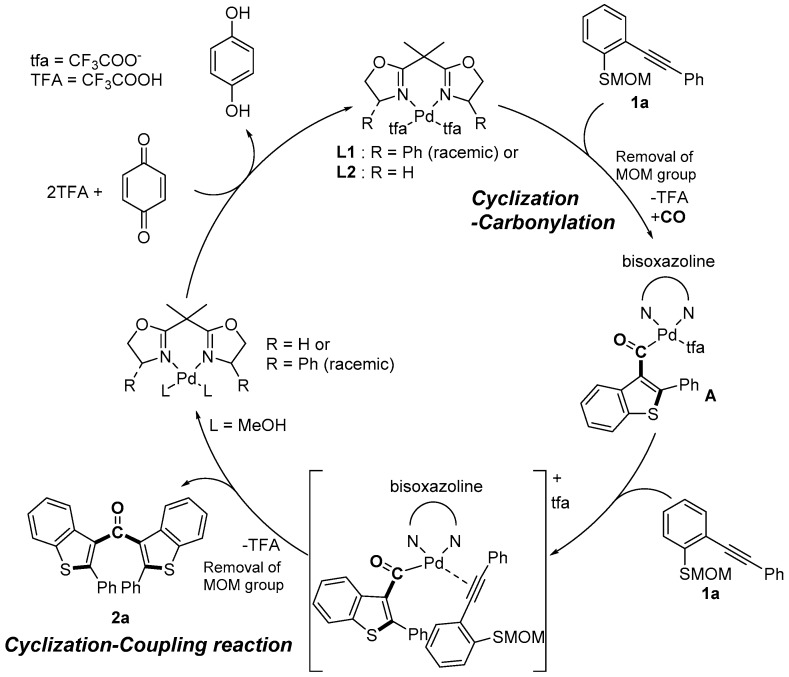
Our previous work: catalytic cycle of cyclization-carbonylation-cyclization coupling reaction (CCC-coupling reaction) reaction. Ph, phenyl; MOM, methoxymethyl.

Cascade reactions are important tools for constructing a variety of heterocycles in one step starting from simple compounds [1,2,3,4]. Recently, we reported that the cyclization-carbonylation-cyclization coupling reaction (CCC-coupling reaction) of (*o*-alkynyl phenyl) (methoxymethyl) sulfides **1** catalyzed by palladium(II)-bisoxazoline (box) complexes afforded bis(benzothiophen-3-yl) methanones **2** in good yield (Scheme 1) [5]. Nucleophilic attack by the sulfur atom at the electrophilically activated triple bond is followed by CO insertion to produce the acyl palladium intermediate **A**. The methoxymethyl group may be removed by acetal exchange (or hydrolysis) during the formation of intermediate **A**. Coordination of the triple bond of a second molecule induces the second cyclization, and reductive elimination then leads to the formation of a ketone bearing two benzothiophene groups. The efficient regeneration of the Pd^II^ species from Pd^0^ is the crucial step for obtaining a high yield of the product, and stoichiometric *p*-benzoquinone was employed as a re-oxidant in this transformation. However, there is a disadvantage to using *p*-benzoquinone: a stoichiometric amount of hydroquinone is formed as unwanted waste. Molecular oxygen is considered an ideal oxidant because it is naturally abundant, inexpensive (or free if used as present in the atmosphere), and environmentally friendly, and does not generate any waste products, thereby fulfilling the requirements of a “green chemistry” reactant [6]. Bäckvall and coworkers have conducted extensive studies of the palladium(II)-mediated oxidative 1,4-addition of nucleophiles to conjugated dienes [7,8]. *p*-Benzoquinone is the most common stoichiometric oxidant used in these reactions, but Bäckvall and coworkers also developed a redox-coupled catalytic system to enable the use of molecular oxygen as the terminal oxidant for aerobic palladium-catalyzed oxidations [9,10,11].

*p*-Benzoquinone and macrocyclic metal complexes are employed in these oxidations as electron-transfer mediators (ETMs). ETMs usually facilitate the oxidation reaction by transporting electrons from the catalyst to the oxidant along a low-energy pathway, thereby increasing the efficiency of oxidation and thus complementing direct oxidation reactions [12]. Herein, we report a Pd^II^/Pd^0^-*p*-benzoquinone/hydroquinone-CuCl_2_/CuCl system that uses environmentally friendly molecular oxygen as the terminal oxidant to catalyze the CCC-coupling reaction of (*o*-alkynyl phenyl) (methoxymethyl) sulfides.

## 2. Results and Discussion

The required starting materials **1** were prepared as described previously [5]. Initially, we selected **1a** as a standard substrate to search for potential co-oxidants. The reaction of **1a** with [Pd(tfa)_2_(L2)] (5 mol %) in methanol under a CO/O_2_ atmosphere (1:1, balloon) generated the dimeric ketone **2a** in 12% yield, along with 2-phenylbenzo[*b*]thiophene **3a** (65% yield) (Table 1, Entry 1). The presence of reduced metal (Pd^0^, black) showed that electron transfer between Pd^0^ and O_2_ is too slow compared with decomposition. Next, five co-oxidants (ETMs) were tested in the reaction (*p-*benzoquinone, CuCl_2_, FeCl_3_·6H_2_O, and VO(acac)_2_; acac, acetyl acetonate) (Table 1, Entries 2–5), of which *p-*benzoquinone and CuCl_2_ gave encouraging but still unacceptable yields (Table 1, Entries 2 and 3). These results suggested that one ETM alone has insufficient oxidation potential to oxidize Pd^0^ by O_2_ and therefore the simultaneous use of two co-oxidants was investigated (Table 1, Entries 6–9). Fortunately, the dimeric ketone **2a** was obtained in 87% yield by using *p*-benzoquinone (10 mol %) and CuCl_2_ (5 mol %) as co-oxidants (Table 1, Entry 6). We next attempted to reduce the amount of *p*-benzoquinone required by investigating the reaction temperature (Table 1, Entries 7–9). The best result was obtained by using *p*-benzoquinone (5 mol %) and CuCl_2_ (5 mol %) as co-oxidants at 0 °C, affording **2a** in 88% yield (Table 1, Entry 8). Having optimized the reaction conditions, we examined the reaction of various (*o*-alkynyl phenyl) (methoxymethyl) sulfide derivatives (Table 2), starting with the reaction of substrates **1b**–**h** bearing aryl substituents at the alkyne terminus (Table 2, Entries 1–8). Neither electron-donating nor electron-withdrawing groups affected the reaction, and **2b**–**d** were obtained in good yield, similar to that of the parent substrate **1a** (Table 2, Entries 2–4). Three different halogen substituents (F, Cl, and Br) and a thiophene ring were tolerated under the reaction conditions used (Table 2, Entries 5–8). Substrates **1i**–**l** bearing alkyl substituents at the alkyne terminus were transformed to the corresponding ketones **2j**–**l** in 71%–92% yield (Table 2, Entries 9–12). Free hydroxyl groups were also tolerated. The scope of the substrate for the CCC-coupling reaction was expanded further by investigating the reactions of substrates bearing R^2^ substituents (Table 2, Entries 13–14). The reactions of **1m**–**o** bearing a Cl substituent, methyl group, and methoxy group in an aromatic moiety proceeded well.

**Scheme 2:**
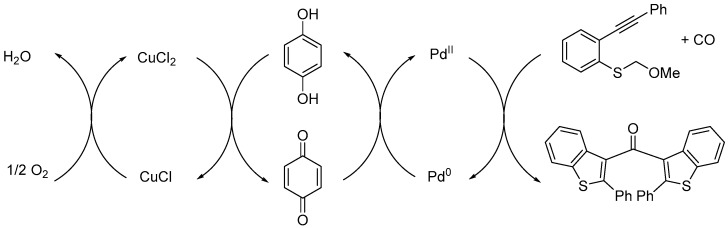
Proposed redox cycles.

This redox-coupled Pd^II^/Pd^0^-*p*-benzoquinone/hydroquinone-CuCl_2_/CuCl triple catalytic system can be described according to Scheme 2. The initial steps of the CCC-coupling reaction are mediated by Pd^II^ [5] and *p*-benzoquinone acts as a co-oxidant (ETM) to transfer protons and electrons from palladium to CuCl_2_. Finally, CuCl is re-oxidized by molecular oxygen, the terminal oxidant [13,14].

**Figure 1:**
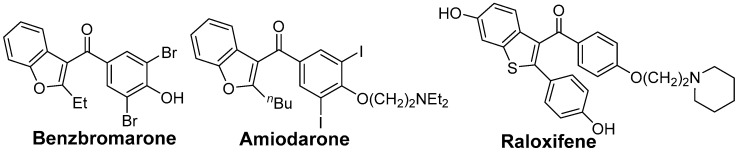
Some drugs having diarylketone scaffolds.

**Scheme 3:**
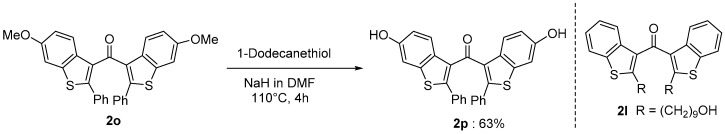
Preparation of **2p** and the structure of **2l**.

**Figure 2:**
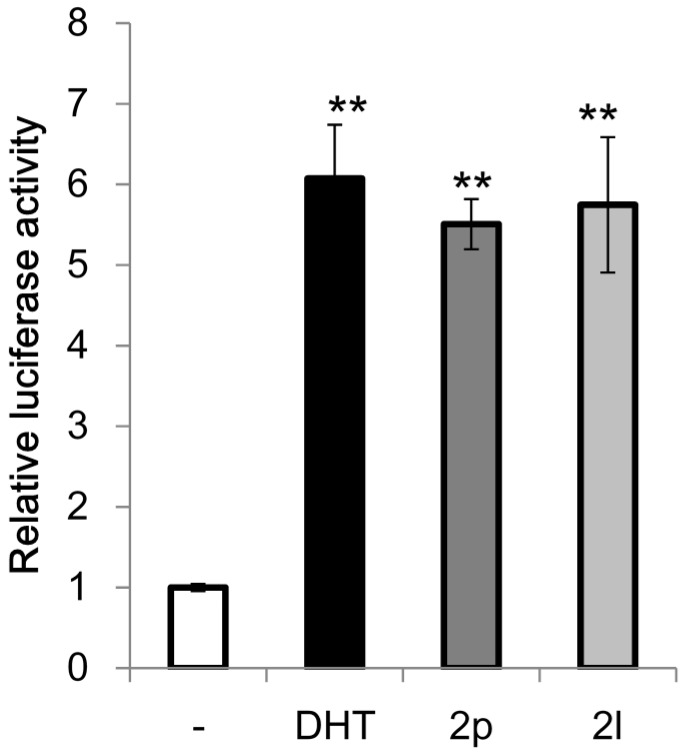
Effect of **2p** and **2l** on ARE-luciferase reporter activity in HEK293 cells. The results are shown as the mean ± S.D. (*n* = 4). ** *p* < 0.01 compared with the solvent control.

Benzo[*b*]thiophene skeletons are an important class of *S*-heterocycles [15,16,17,18] and are found in a variety of drugs, pesticides, and biologically active compounds that exhibit various interesting biological properties [19,20,21,22,23,24]. Diaryl ketone scaffolds are also important motifs in natural products and pharmaceuticals [25,26,27,28,29,30] (Figure 1). Androgens are known to have beneficial anabolic actions on various tissues such as bone and muscle. However, the clinical use of androgens has been limited because of their undesirable sexually actions. Recently, non-steroidal androgens have been investigated in many laboratories. As a preliminary study, we tested the androgen receptor (AR) agonistic activity of **2p** and **2l**. Demethylation of **2o** afforded **2p** in 63% yield (Scheme 3). We performed androgen response element (ARE)-driven luciferase reporter assay (ARE-luc.) in human kidney derived HEK293 cells. A portion of 10 μM of **2p** and **2l** were examined for their ability to activate the transcription of the ARE-luc. reporter gene (Figure 2). Both **2p** and **2l** elicited ARE-luciferase reporter activity similarly to dihydrotestosterone (DHT, 10 nM). This observation suggested that dibenzo[*b*]thiophenyl ketone scaffolds (such as **2p** and **2l**) may expect to be pharmacophores for non-steroidal AR-agonist. We are currently investigating further biological studies of the synthesized compounds **2**.

The HEK293 cells were transfected with the ARE-luciferase reporter, AR expression, and pGL4.74 plasmids. The next day, cells were treated with dihydrotestosterone (DHT) (10 nM), **2p** (10 μM), or **2l** (10 μM) for 24 h. Luciferase activity was measured using the Dual-Luciferase Reporter Assay System. The results are shown as the mean ± S.D. (*n* = 4). Statistically significant differences were determined using one-way analysis of variance followed by Dunnett’s multiple comparison test as the post-hoc test.

## 3. Experimental

### 3.1. General Information

^1^H and ^13^C-NMR spectra was recorded on JEOL ECS 400 (JEOL, Tokyo, Japan) and JEOL ECA 500 spectrometers (JEOL, Tokyo, Japan) in CDCl_3_ with Me_4_Si as an internal reference. When the solvent was DMSO-*d*_6_, solvent peak was used as a reference (2.50 ppm for ^1^H, and 39.5 ppm for ^13^C). ^13^C-NMR spectra were recorded at 100 MHz. All reagents were purchased from commercial sources and used without purification. All evaporations were performed under reduced pressure. Silica gel (Kieselgel 60, Merck, Kenilworth, NJ, USA) was used for column chromatography. CO and O_2_ were measured and injected into a balloon using a jumbo syringe (SGE Analytical Science, Milton Keynes, UK).

### 3.2. Preparation of Substrates

The (*o*-alkynyl phenyl) (methoxymethyl) sulfides **1a**–**n** were prepared from known *o*-iodoanilines using a published procedure, and the spectral data were identical to those described in the literature [5].

### 3.3. General Procedure for the Reaction of (o-Alkynyl Phenyl) (Methoxymethyl) Sulfides ***1***

A 30 mL two-necked round-bottom flask containing a magnetic stir bar, (*o*-alkynyl phenyl) (methoxymethyl) sulfide **1** (0.4 mmol), *p*-benzoquinone (2.2 mg, 0.02 mmol), CuCl_2_ (3.4 mg, 0.02 mmol), and MeOH (3 mL) was fitted with a rubber septum and a three-way stopcock connected to a balloon filled with CO and O_2_ (500 mL:500 mL). The apparatus was purged with the gas from the balloon by pump-filling via the three-way stopcock. A MeOH (1 mL) suspension of [Pd(tfa)_2_(**L2**)] (10.3 mg, 0.02 mmol) was added to the stirred solution at an appropriate temperature via a syringe. The residual catalyst was washed with MeOH (1 mL) twice, and the reaction mixture was stirred for 24–72 h. In most cases, the dimeric ketones **2** precipitated from the reaction mixture. The resulting precipitate was collected by filtration and washed with cold MeOH (1.5 mL × 2) to yield dimeric ketones **2**. The small amount of **2** remaining in the filtrate was recovered by diluting the filtrate with CH_2_Cl_2_ (50 mL) and washing with 5% NaOH (40 mL). The aqueous layer was extracted with CH_2_Cl_2_ (25 mL) and the combined organic layers were dried over MgSO_4_ and concentrated in vacuo. The crude product was purified by column chromatography on silica gel. The fraction eluted with hexane/EtOAc (100:1) afforded small amounts of dimeric ketones **2**. The spectral data of products **2a**–**o** were identical to those described in the literature [5].

#### Preparation of bis(6-hydroxy-2-phenylbenzo*[b]*thiophen-3-yl)methanone, **2p**

To a suspension of sodium hydride (58 mg, 1.2 mmol, 50% in mineral oil) and 1-dodecanethiol (243 mg, 1.2 mmol) in anhydrous DMF (5 mL) under Ar was added **2o** (101.2 mg, 0.2 mmol), and the mixture was heated at 110 °C for 4 h. The mixture was allowed to cool, and was then diluted with ice-water. The mixture was extracted with CH_2_Cl_2_ (30 mL) twice. The combined organic layers were dried over MgSO_4_ and concentrated in vacuo. The crude product was purified by column chromatography on silica gel. The fraction eluted with EtOAc afforded **2p** (60 mg, 63% yield) as white solid.mp: 259–260 °C; ^1^H-NMR (DMSO-*d*_6_): δ 6.89–6.97 (8H, m), 7.03 (2H, dd, *J* = 2.4, 8.0 Hz), 7.08–7.14 (4H, m), 7.98 (2H, d, *J* = 8.8 Hz), 9.79 (2H, s); ^13^C-NMR (DMSO-*d*_6_): δ 106.7 (2C), 115.7 (2C), 124.3 (2C), 127.6 (4C), 128.4 (2C),128.5 (4C), 132.1 (2C), 132.3 (2C), 132.5 (2C), 139.2 (2C), 146.4 (2C), 155.5 (2C), 188.5; IR (KBr): 3651, 2925, 1734, 1617, 1560, 1541, 1523, 1508 cm^−1^; HRMS-EI: *m*/*z*: [M+] calcd for C_29_H_18_O_3_S_2_ 478.0697 found 478.0696 (See Supplementary Materials for more details).

### 3.4. Agonistic Activity of ***2p*** and ***2l***

The cells were seeded in 48-well plates and transfected with appropriate expression plasmids, the ARE-luciferase reporter plasmid, AR expression plasmid, and a Renilla pGL4.74 [hRluc/TK] (Promega, Madison, WI, USA) as an internal standard by the reverse-transfection method using the PEI Max reagent (Polysciences Inc., Warrington, PA, USA). After overnight incubation in phenol red-free DMEM containing 5% charcoal-stripped FBS (Promega, Madison, WI, USA), the cells were treated with various compounds for 24 h before luciferase activity was measured using the Dual-Luciferase Reporter Assay System (Promega, Madison, WI, USA). Firefly luciferase activities were normalized against Renilla luciferase activities.

## 4. Conclusions

In summary, we developed a multistep electron-transfer process involving a “triple-catalysis” system: a Pd^II^/Pd^0^-*p*-benzoquinone/hydroquinone-CuCl_2_/CuCl catalytic system that uses environmentally friendly molecular oxygen as the terminal oxidant to effectively catalyze the CCC-coupling reaction of (*o*-alkynyl phenyl) (methoxymethyl) sulfides **2**. Synthesized compounds **2p** and **2l** showed the androgen receptor (AR) agonistic activity. Dibenzo[*b*]thiophenyl ketone scaffold may expect to pharmacophore for non-steroidal AR-agonist. We are currently investigating further biological studies of the synthesized compounds **2**.

## Figures and Tables

**Table 1:**
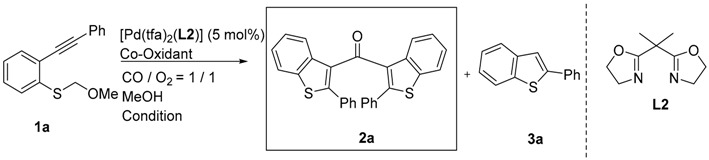
Optimization of the CCC-coupling reaction of **1a**.

Entry	Co-Oxidant	Conditions	Yield of 2 (%)	Yield of 3 (%)	Recovery (%)
1	None	15 °C ~rt, 72 h	12	65	trace
2	*p-*BQ (10 mol %)	5 °C ~rt, 72 h	48	19	-
3	CuCl_2_ (5 mol %)	5 °C, 24 h	46	16	14
4	FeCl_3_·6H_2_O (5 mol %)	5 °C, 24 h	7	-	87
5	VO(acac)_2_ (5 mol %)	5 °C, 24 h	31	12	32
6	*p-*BQ (10 mol %) CuCl_2_ (5 mol %)	5 °C, 24 h	87	8	-
7	*p-*BQ (5 mol %) CuCl_2_ (5 mol %)	5 °C, 24 h	84	7	-
8	***p-*BQ (5 mol %)** **CuCl_2_ (5 mol %)**	**0 °C, 48 h**	**88**	**5**	**-**
9	*p-*BQ (5 mol %) CuCl_2_ (5 mol %)	−5 °C, 48 h	65	trace	20

**Table 2:**
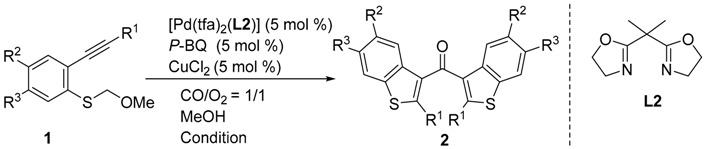
Scope of suitable substrates for the CCC-coupling reaction.

Entry	R^1^	R^2^	R^3^	Substrate	Conditions	Yield of 2 (%)
1	Ph	H	H	**1a**	0 °C, 48 h	**2a**: 88
2	4-MePh	H	H	**1b**	−10 °C, 48 h	**2b**: 84
3	4-MeOPh	H	H	**1c**	−10 °C, 48 h	**2c**: 93
4	4-CF_3_Ph	H	H	**1d**	0 °C, 48 h	**2d**: 83
5	4-FPh	H	H	**1e**	−10 °C, 48 h	**2e**: 80
6	4-BrPh	H	H	**1f**	−10 °C, 48 h	**2f**: 82
7	4-ClPh	H	H	**1g**	−10 °C, 48 h	**2g**: 82
8	3-Thienyl	H	H	**1h**	0 °C, 48 h	**2h**: 80
9	Phenethyl	H	H	**1i**	0 °C, 48 h	**2i**: 92
10	Octyl	H	H	**1j**	−10 °C, 48 h	**2j**: 71
11	Cyclopropyl	H	H	**1k**	−10 °C, 48 h	**2k**: 90
12	(CH_2_)_9_OH	H	H	**1l**	−10 °C, 48 h	**2l**: 84
13	Ph	Cl	H	**1m**	−10 °C, 72 h	**2m**: 80
14	Ph	Me	H	**1n**	−10 °C, 48 h	**2n**: 96
15	Ph	H	OMe	**1o**	−10 °C, 48 h	**2o**: 82

## Supplementary Materials

The following are available online at http://www.mdpi.com/1420-3049/21/9/1177/s1. ^1^H and ^13^C-NMR spectra of the synthesized compounds.
