# Supplementary material for: Palladium(II) Catalyzed Cyclization-Carbonylation-Cyclization Coupling Reaction of (ortho-Alkynyl Phenyl) (Methoxymethyl) Sulfides Using Molecular Oxygen as the Terminal Oxidant
Source: Molecules. 2016 Sep 5;21(9):1177. doi: 10.3390/molecules21091177 (PMC6273608; doi:10.3390/molecules21091177)
Supplement: Supplementary file 1 [file molecules-21-01177-s001.pdf]

Supplementary Materials: Palladium(II) Catalyzed Cyclization–Carbonylation–Cyclization Coupling Reaction of (*ortho*-Alkynyl Phenyl) (Methoxymethyl) Sulfides using Molecular Oxygen as the Terminal Oxidant

Rong Shen, Taichi Kusakabe, Tomofumi Yatsu, Yuichiro Kanno, Keisuke Takahashi, Kiyomitsu Nemoto and Keisuke Kato*

General Information

^{1}H and ^{13}C-NMR spectra were recorded on JEOL ECS 400 (JEOL, Tokyo, Japan) in $CDCl_3$ with Me_4Si as an internal reference. When the solvent was DMSO-d_6, solvent peaks were used as a reference (2.50 ppm for ^{1}H, and 39.5 ppm for ^{13}C). ^{13}C-NMR spectra were recorded at 100 MHz. All reagents were purchased from commercial sources and used without purification. All evaporations were performed under reduced pressure. Silica gel (Kieselgel 60, Kenilworth, NJ, USA) was used for column chromatography. CO and O_2 were measured and injected into a balloon using a jumbo syringe (SGE Analytical Science, Milton Keynes, UK).

^{1}H and ^{13}C-NMR spectra

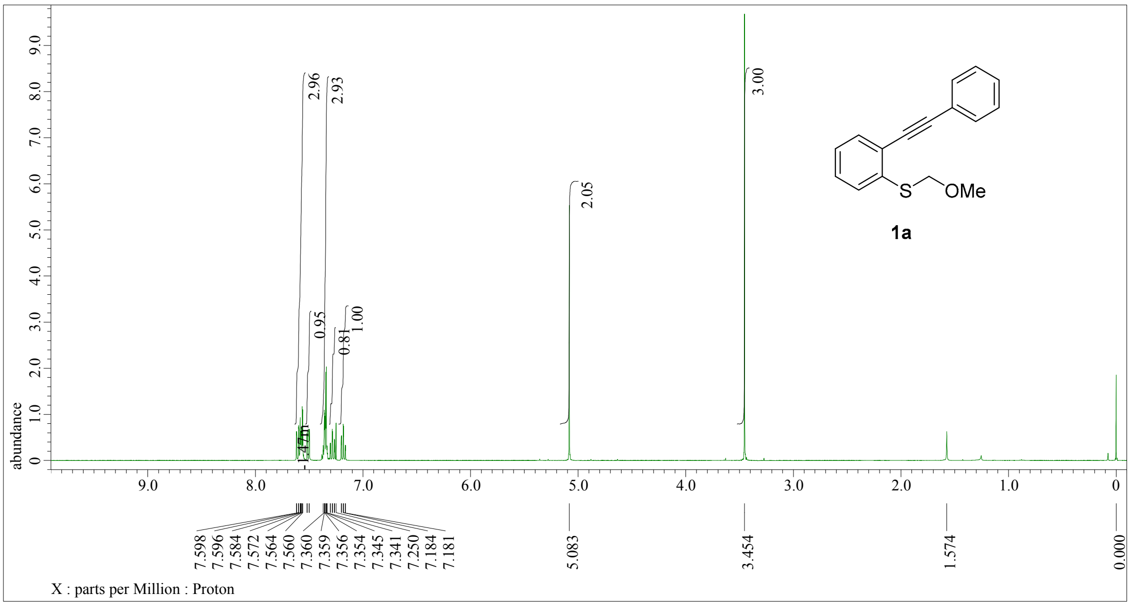

Figure S1. ^{1}H-NMR of compound **1a**.

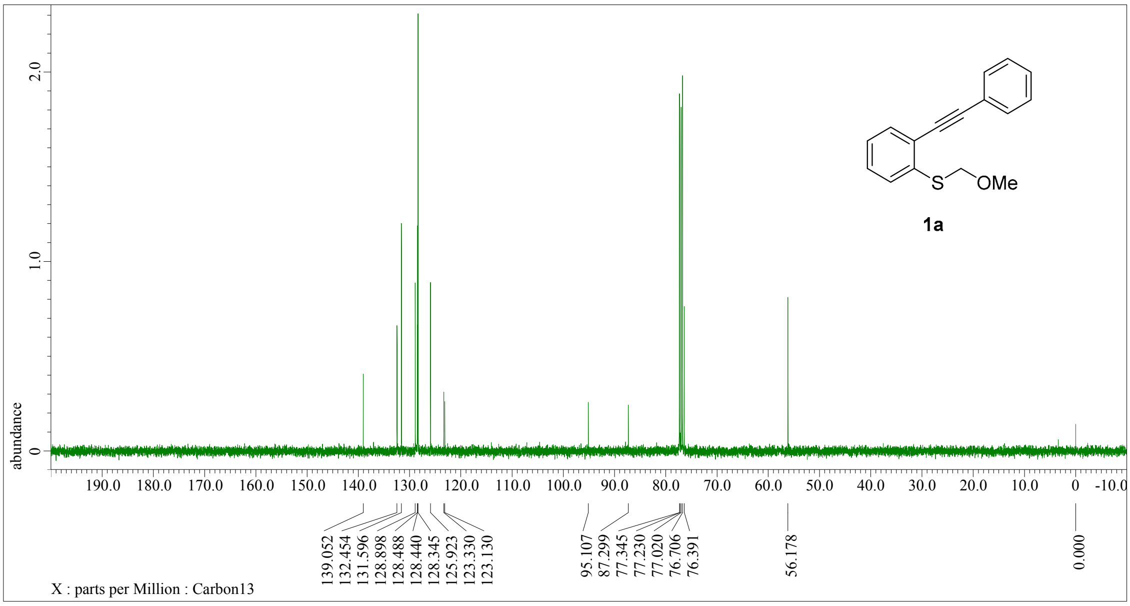

Figure S2. ^{13}C-NMR of compound **1a**.

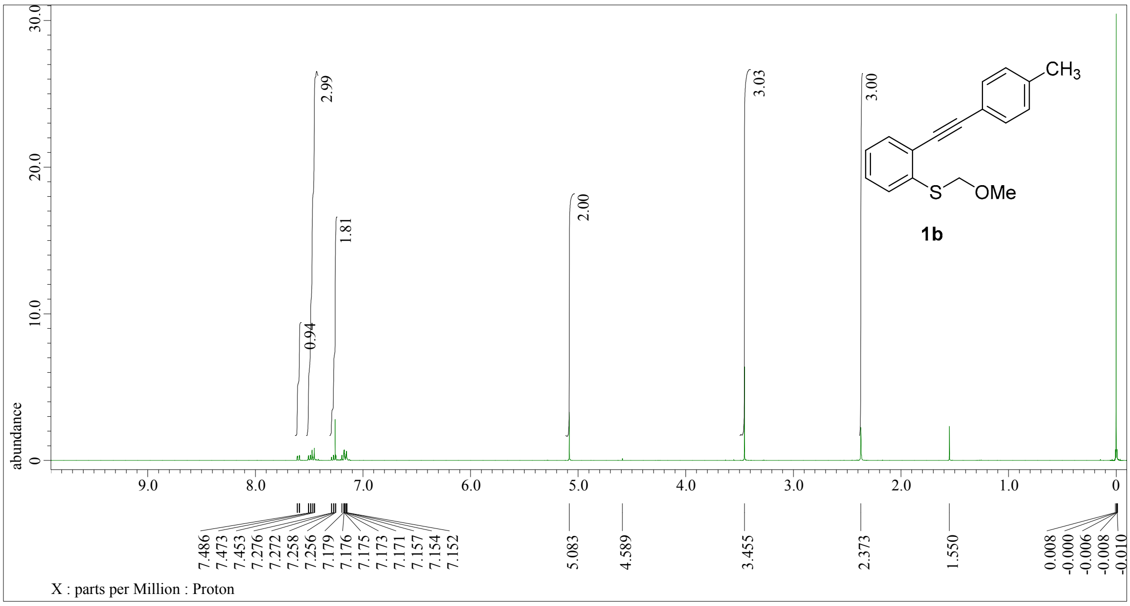

Figure S3. ^{1}H-NMR of compound **1b**.

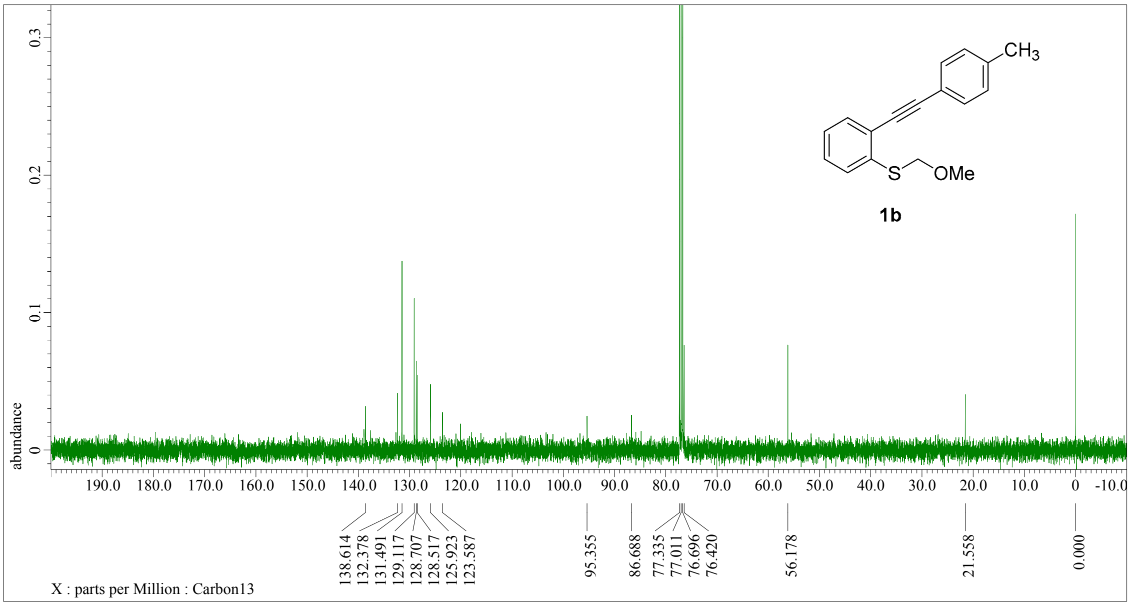

Figure S4. ^{13}C-NMR of compound **1b**.

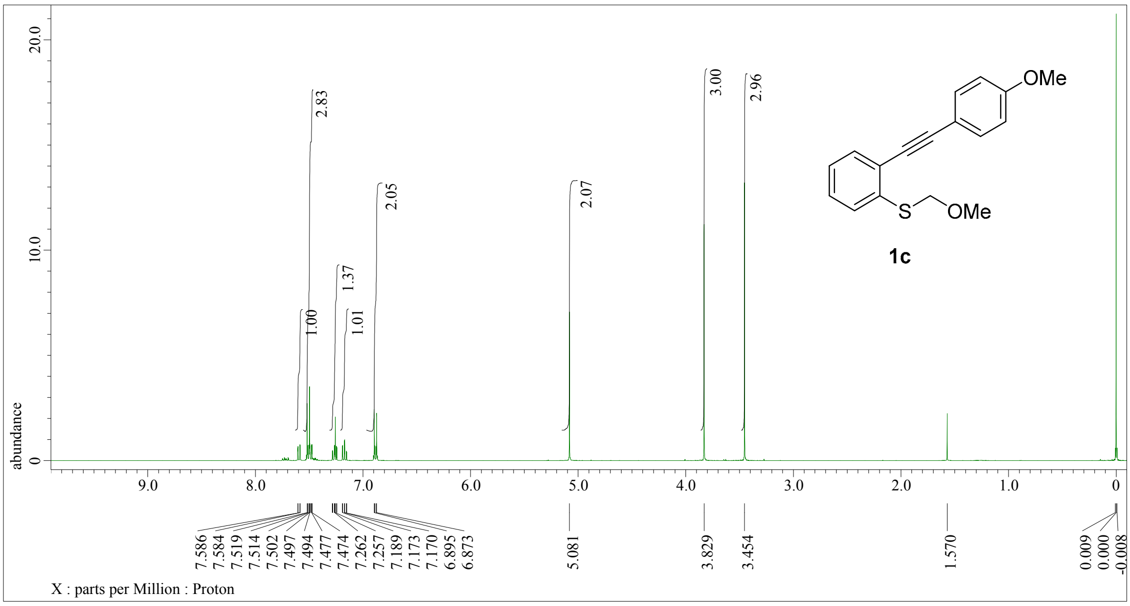

Figure S5. ^{1}H-NMR of compound **1c**.

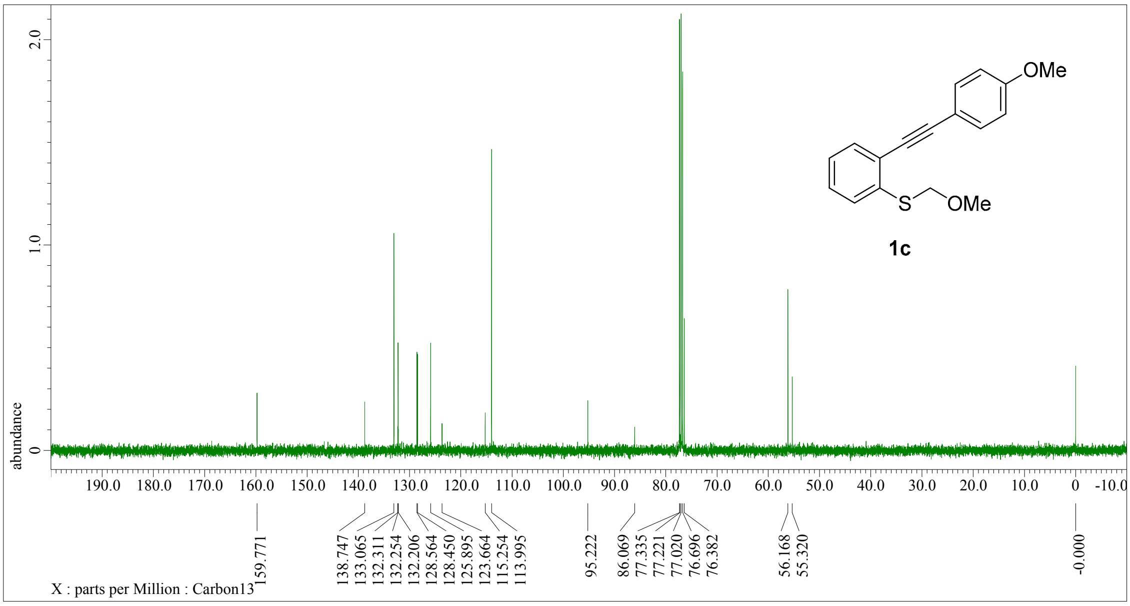

Figure S6. ^{13}C-NMR of compound **1c**.

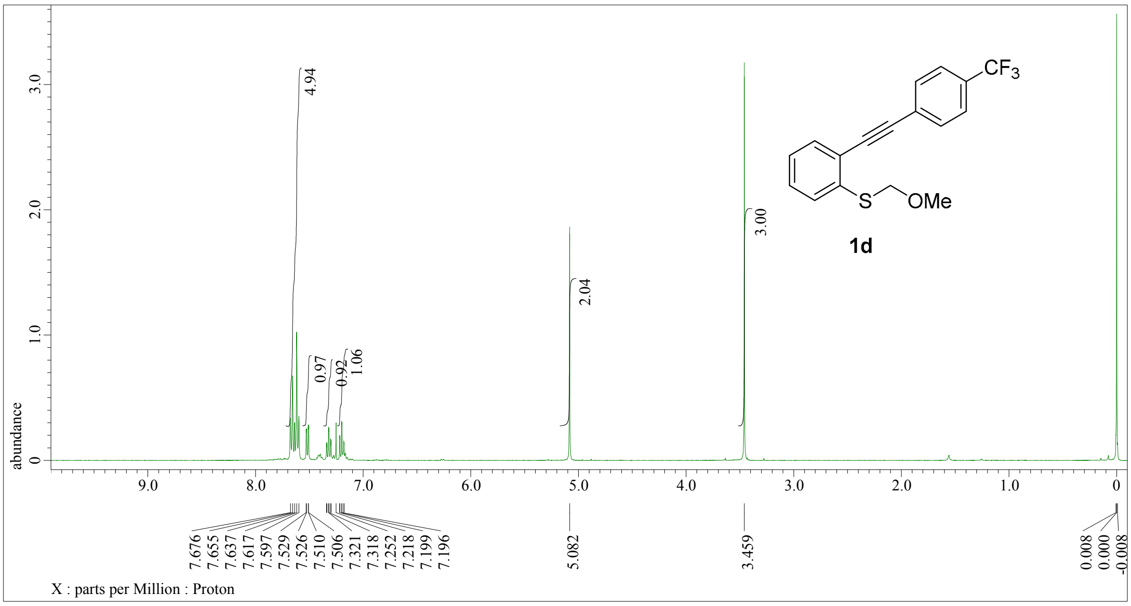

Figure S7. ^{1}H-NMR of compound **1d**.

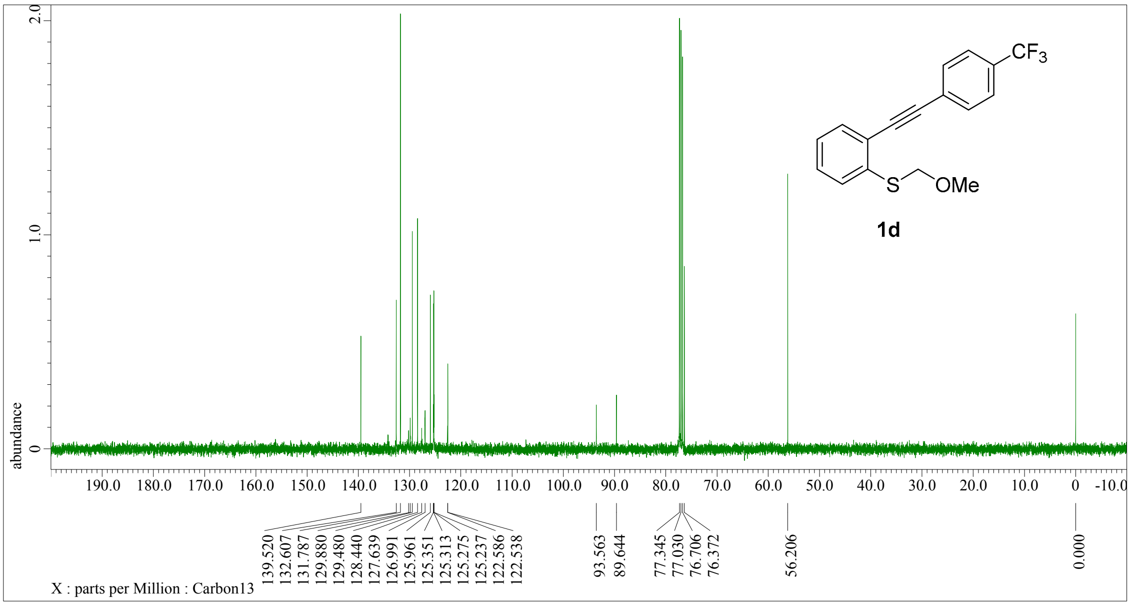

Figure S8. ^{13}C-NMR of compound **1d**.

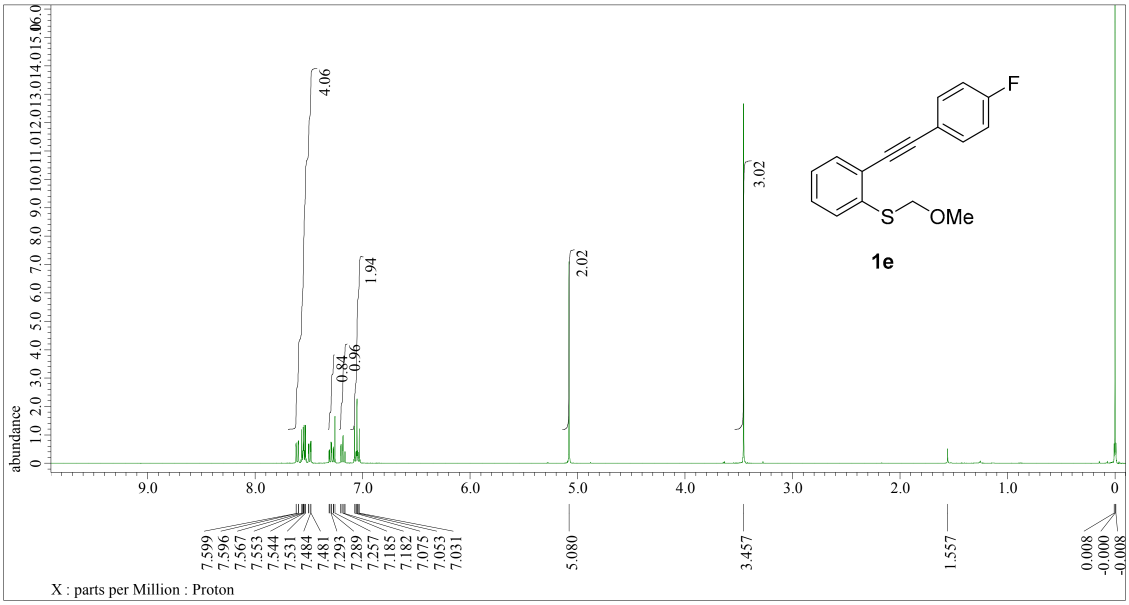

Figure S9. ^{1}H-NMR of compound **1e**.

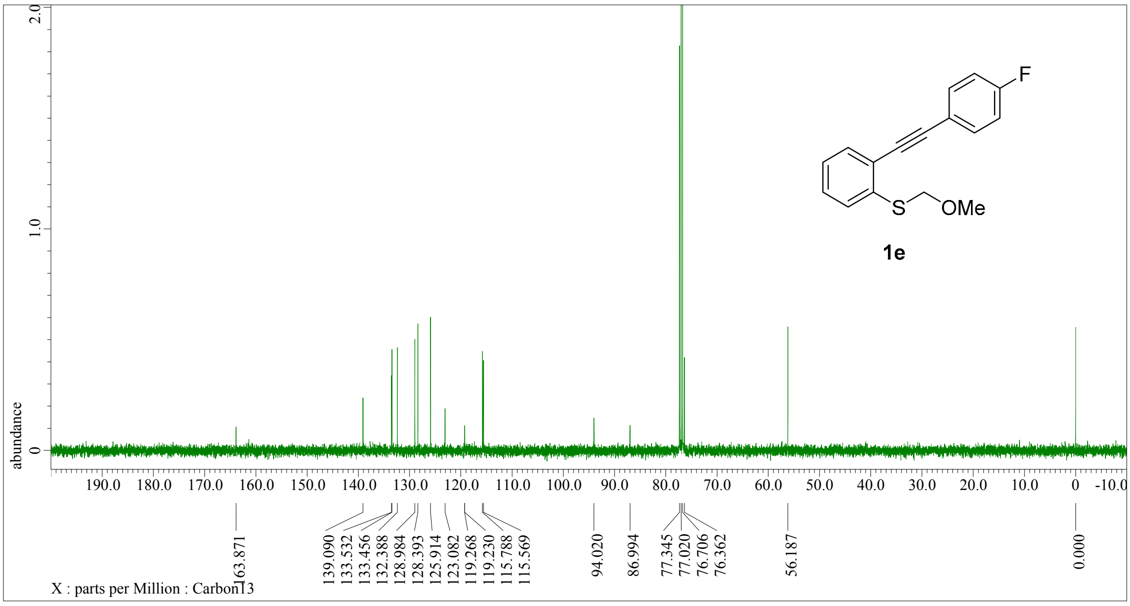

Figure S10. ^{13}C-NMR of compound **1e**.

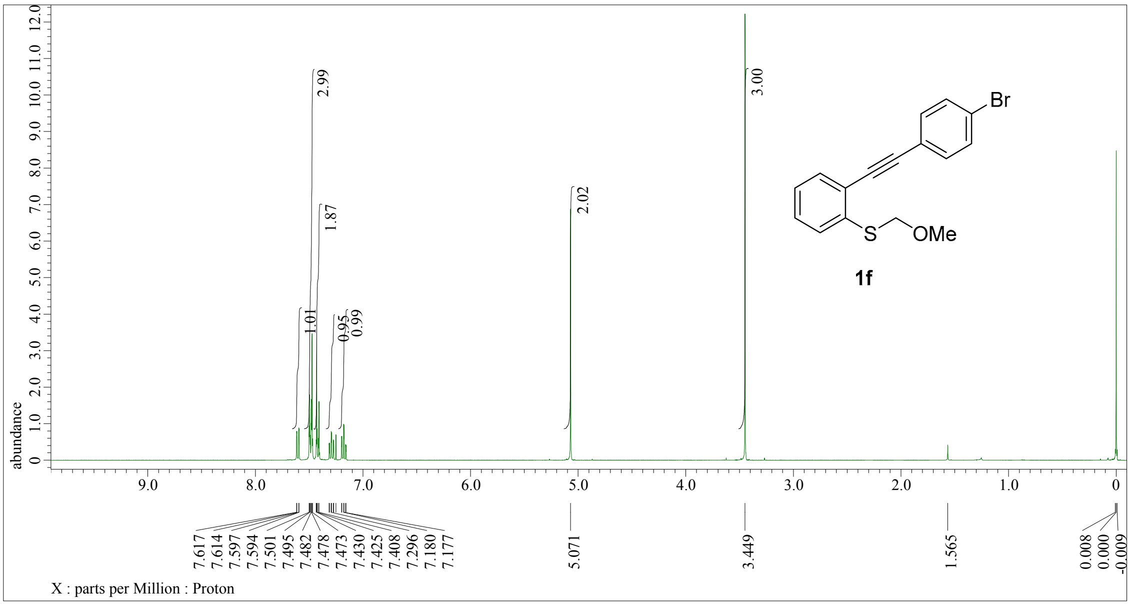

Figure S11. ^{1}H-NMR of compound **1f**.

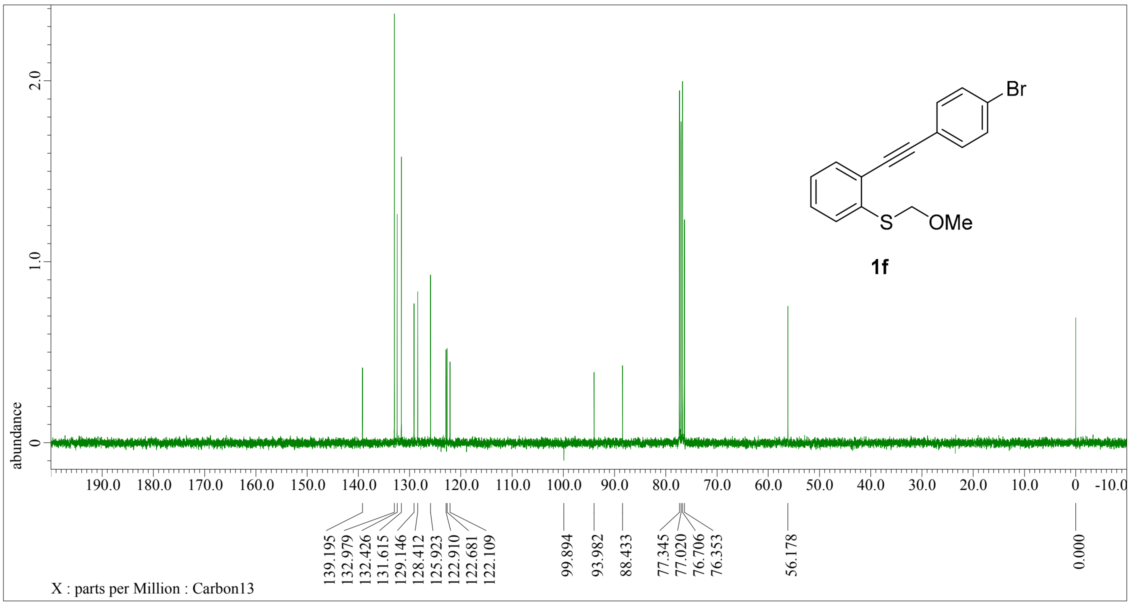

Figure S12. ^{13}C-NMR of compound **1f**.

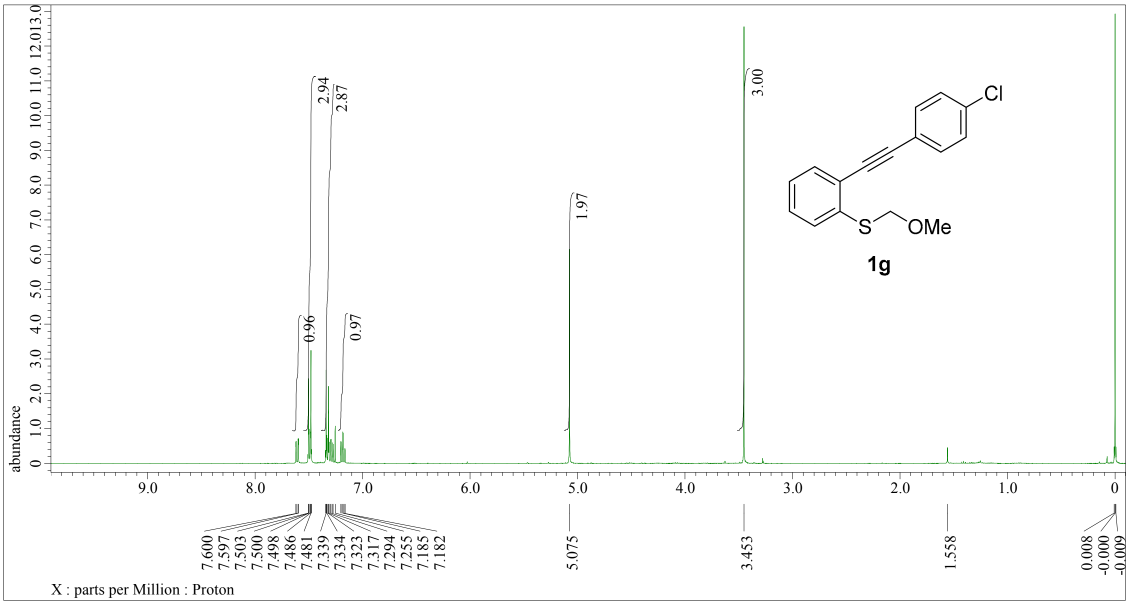

Figure S13. ^{1}H-NMR of compound **1g**.

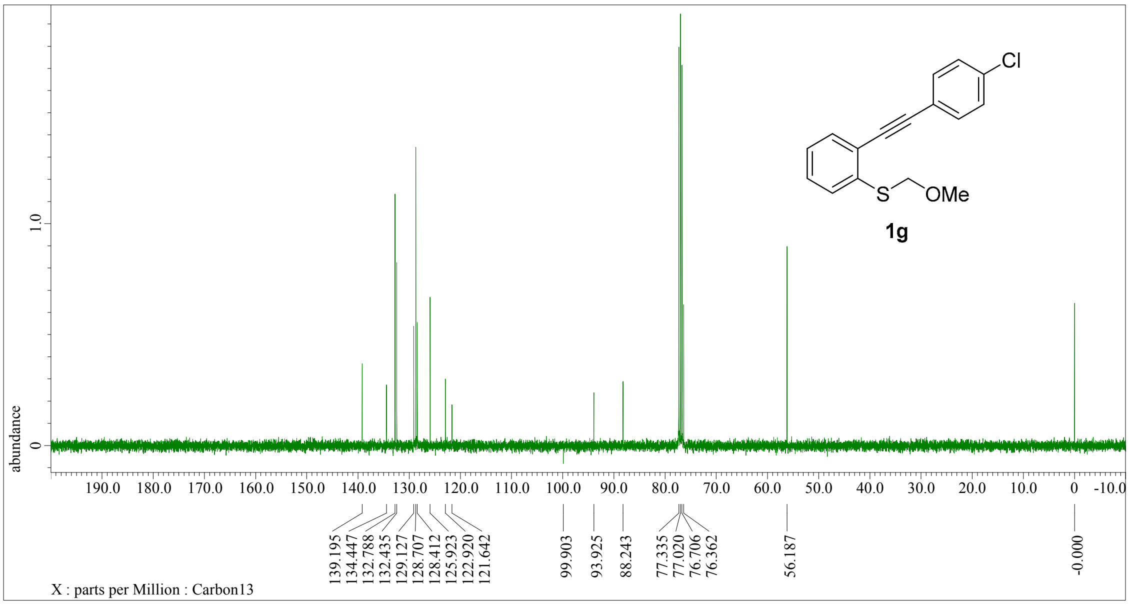

Figure S14. ^{13}C-NMR of compound **1g**.

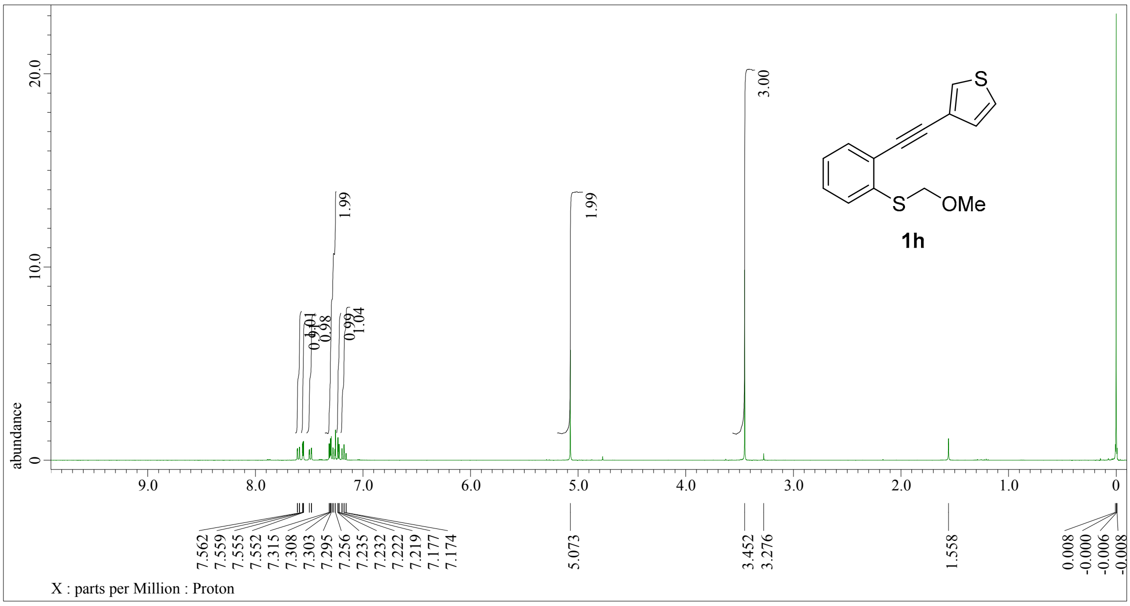

Figure S15. ^{1}H-NMR of compound **1h**.

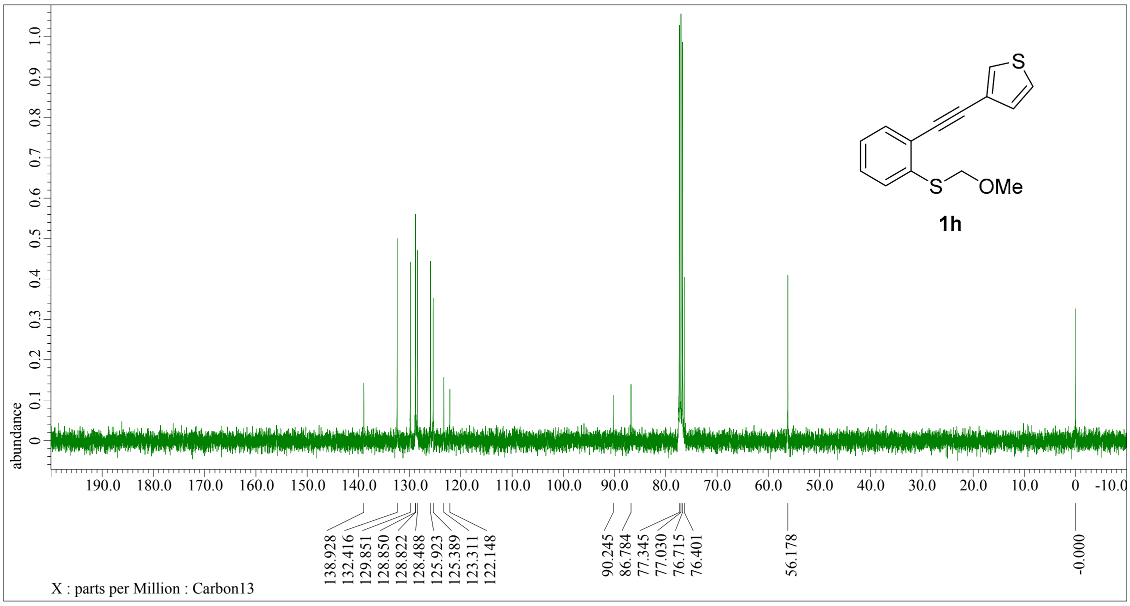

Figure S16. ^{13}C-NMR of compound **1h**.

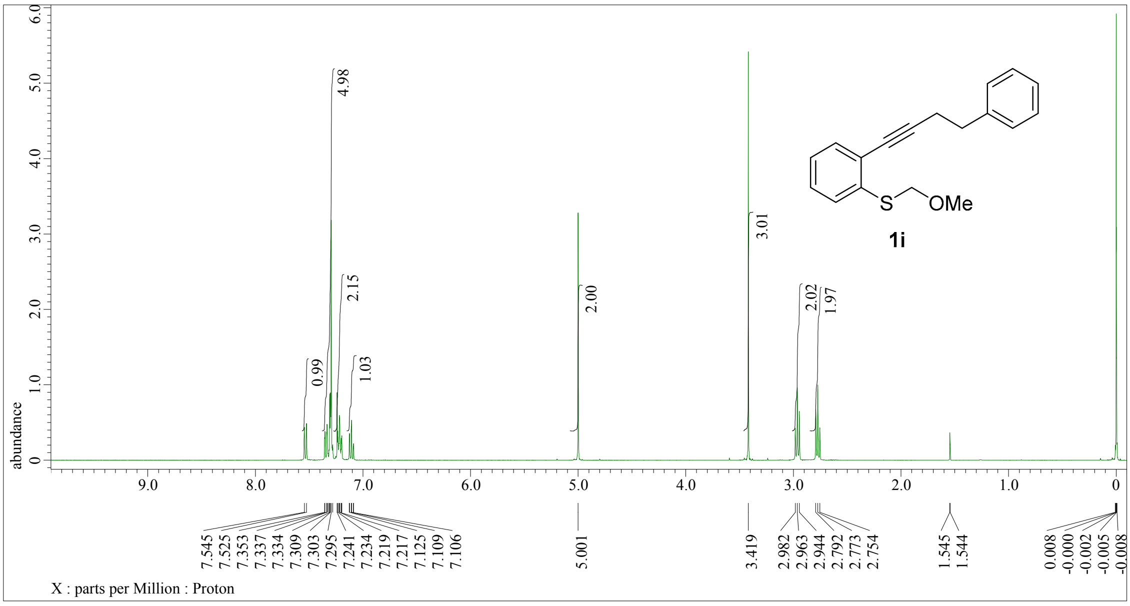

Figure S17. ^{1}H-NMR of compound **1i**.

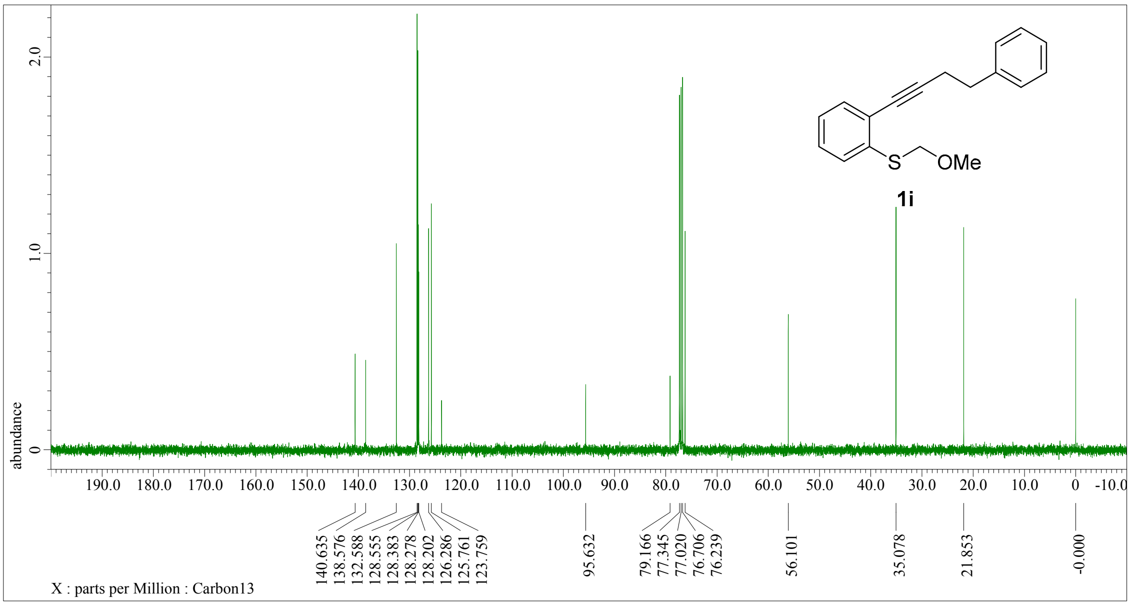

Figure S18. ^{13}C-NMR of compound **1i**.

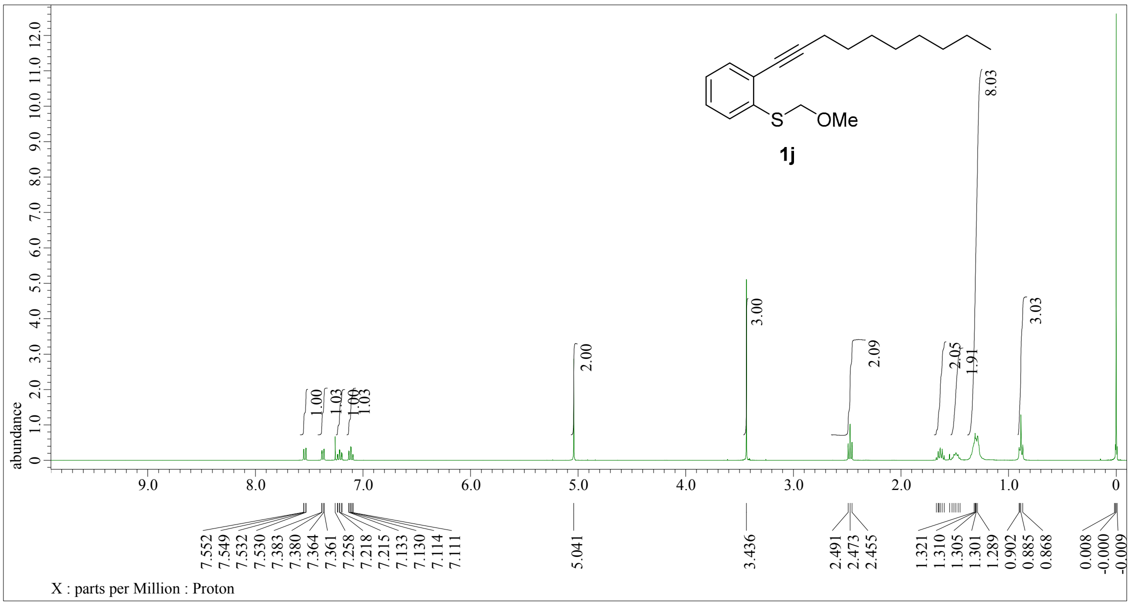

Figure S19. ^{1}H-NMR of compound **1j**.

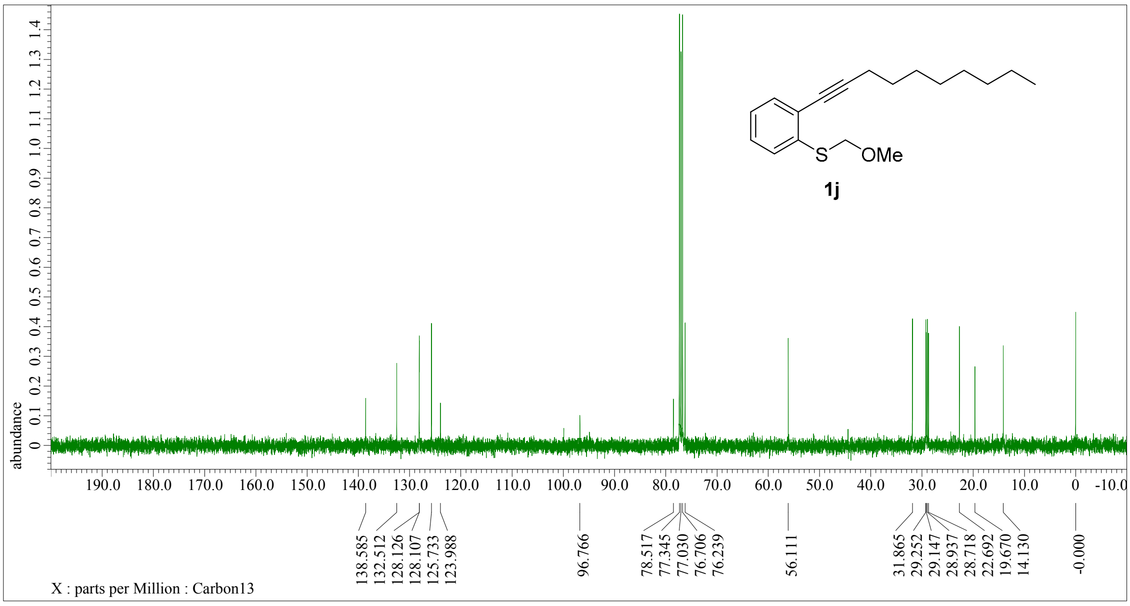

Figure S20. ^{13}C-NMR of compound **1j**.

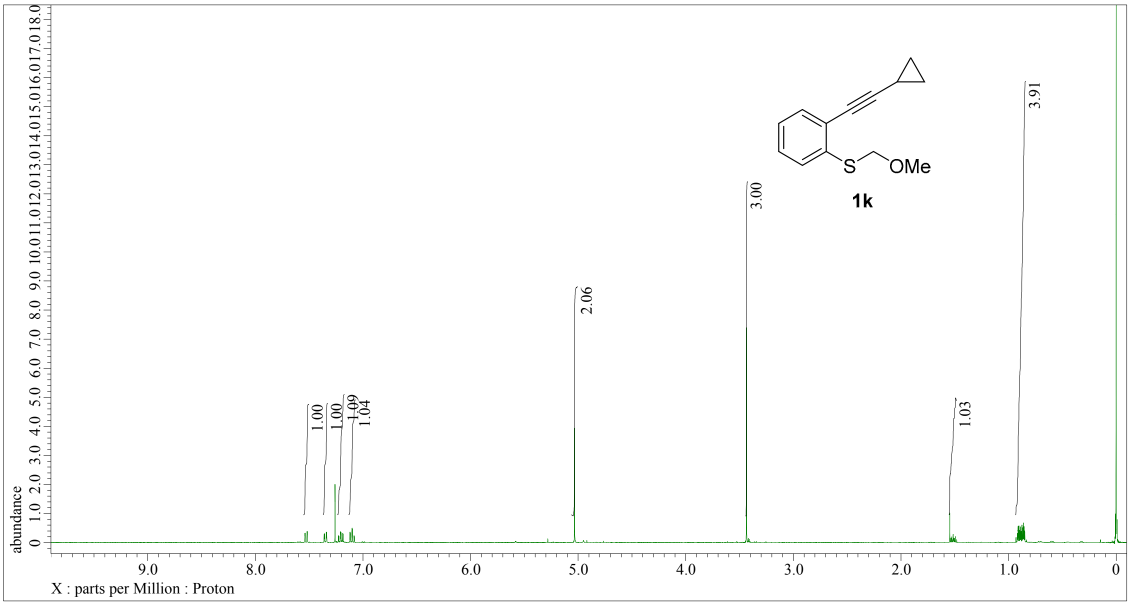

Figure S21. ^{1}H-NMR of compound **1k**.

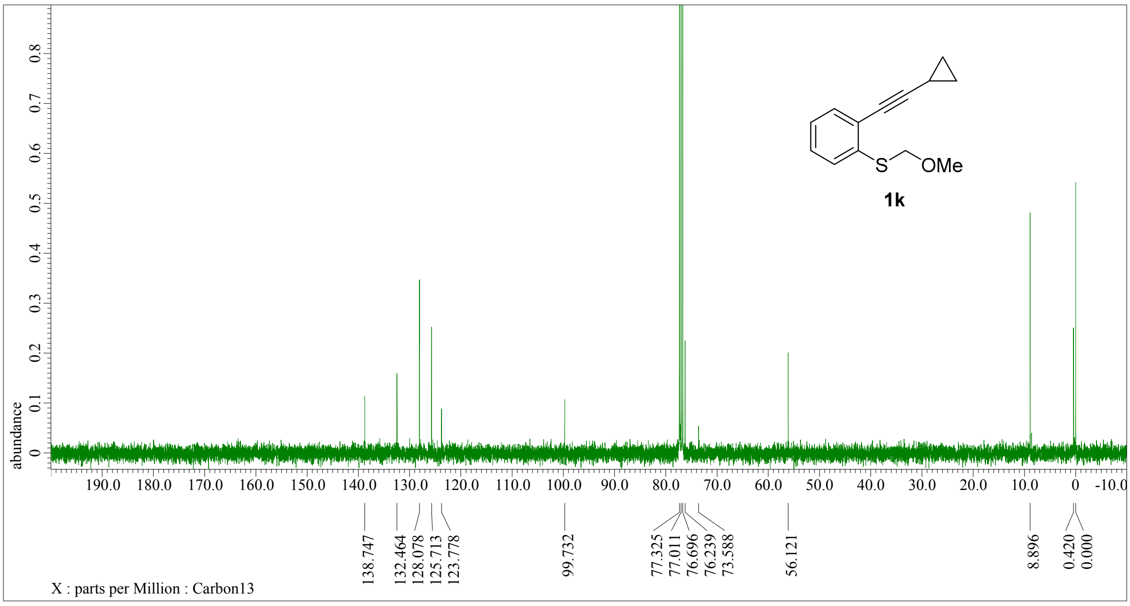

Figure S22. ^{13}C-NMR of compound **1k**.

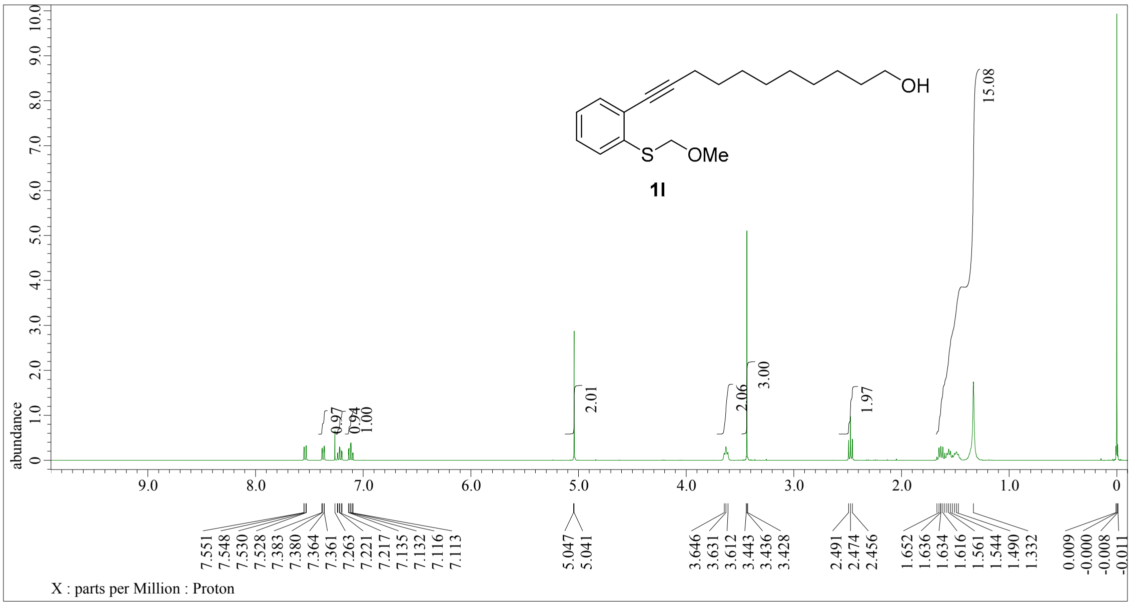

Figure S23. ^{1}H-NMR of compound **1l**.

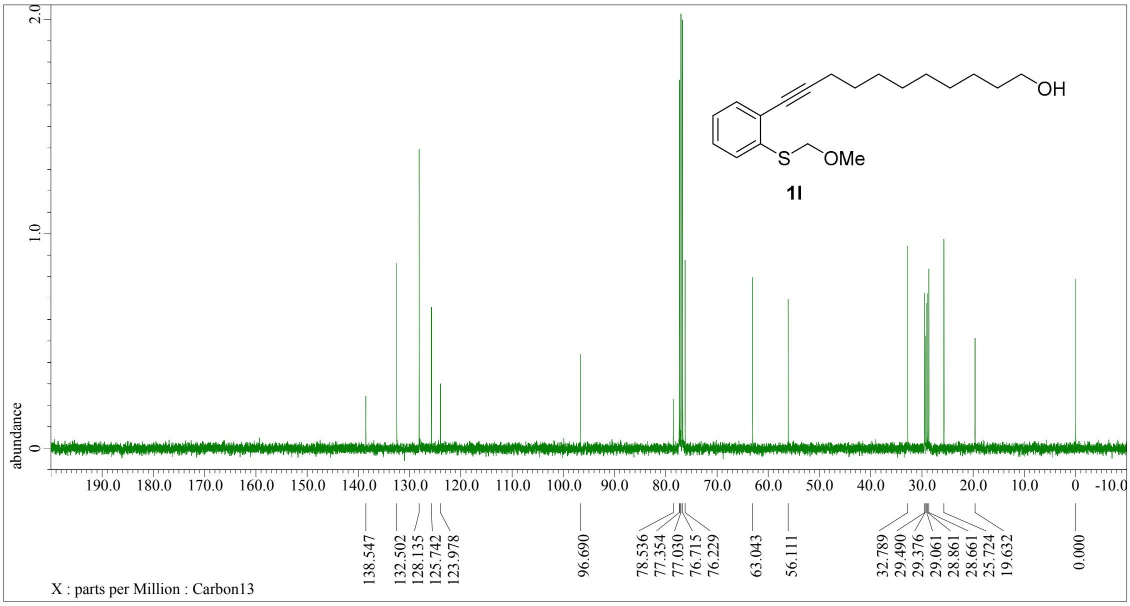

Figure S24. ^{13}C-NMR of compound **1l**.

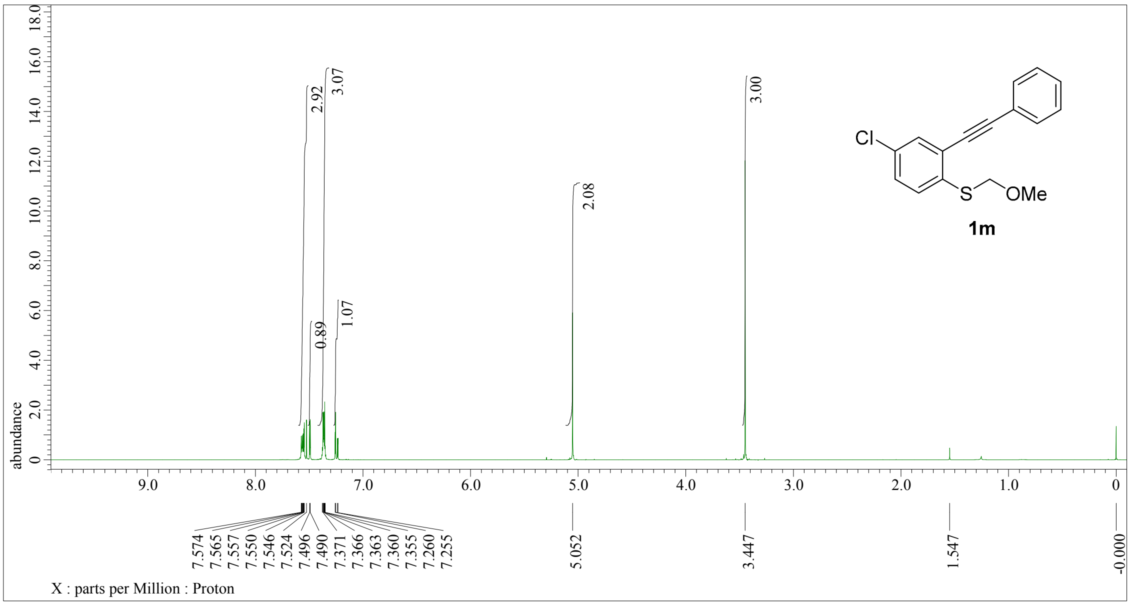

Figure S25. ^{1}H-NMR of compound **1m**.

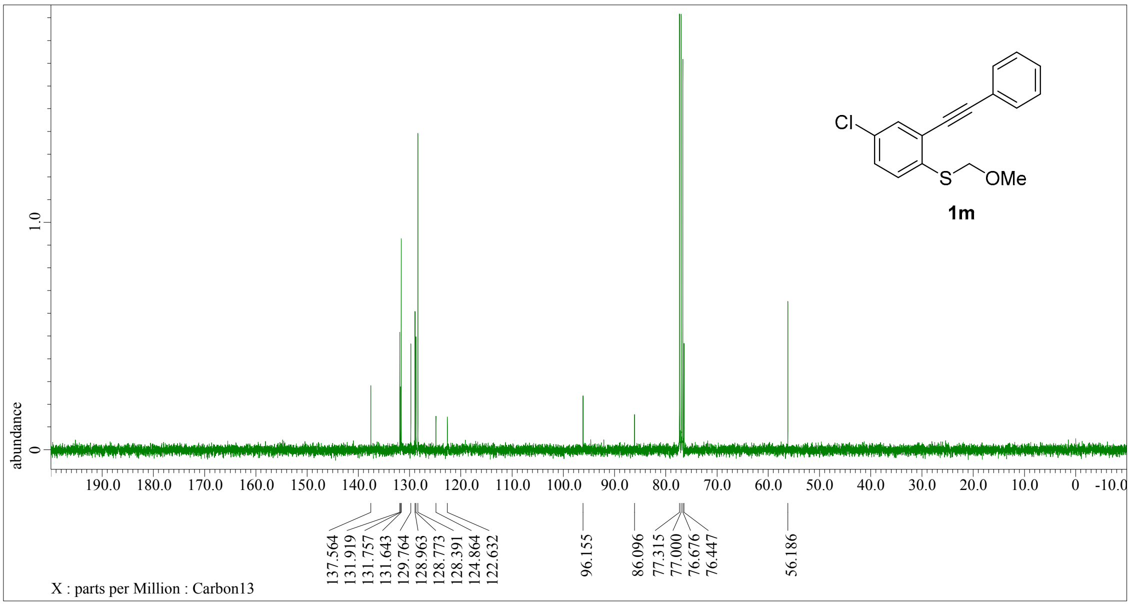

Figure S26. ^{13}C-NMR of compound **1m**.

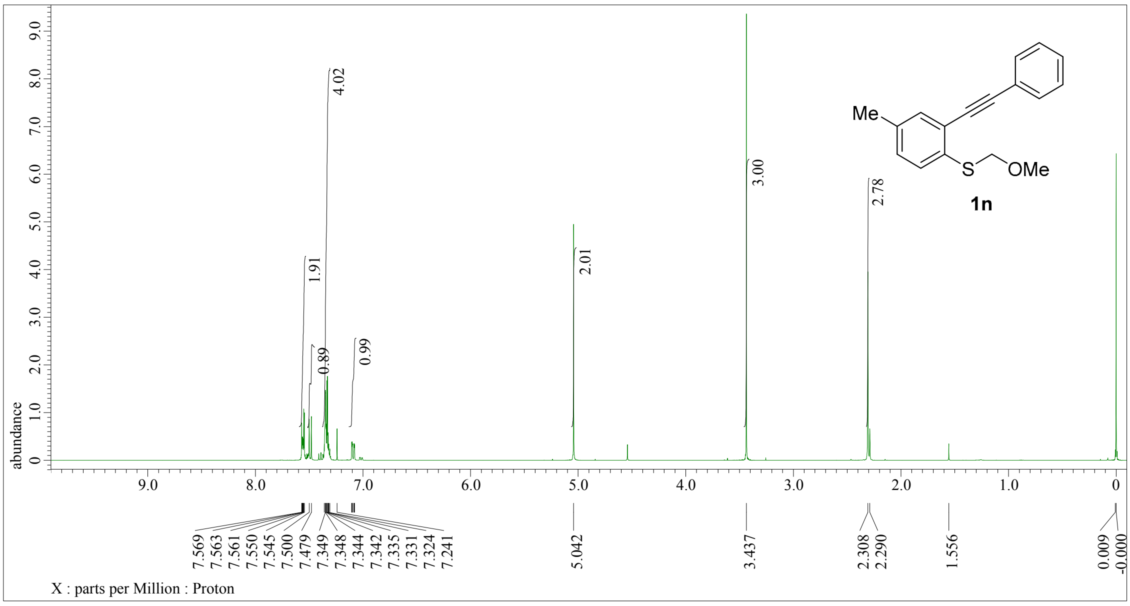

Figure S27. ^{1}H-NMR of compound **1n**.

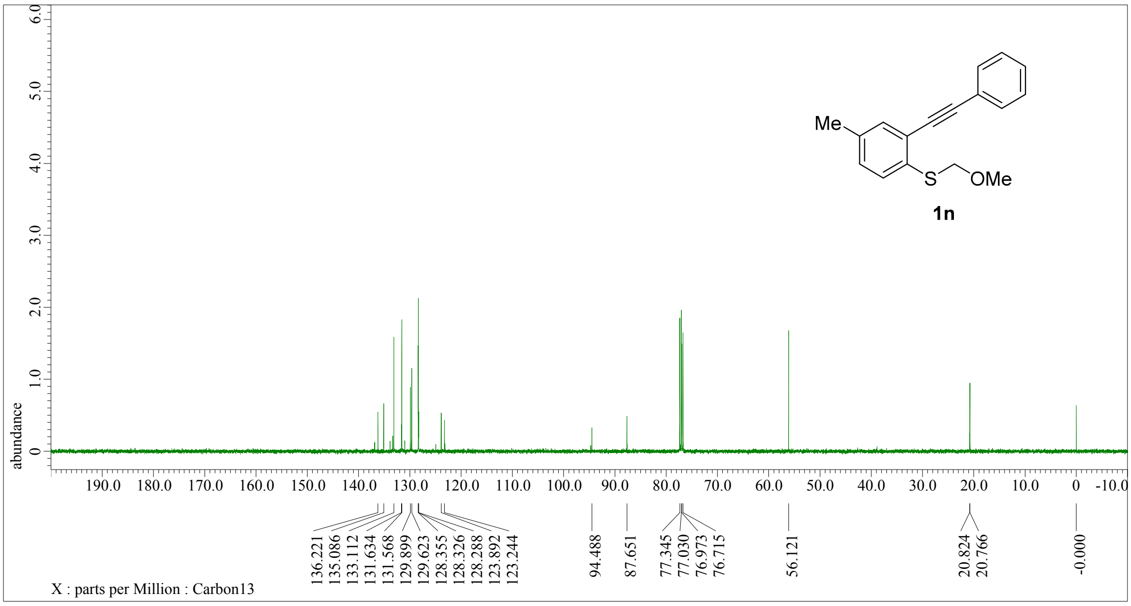

Figure S28. ^{13}C-NMR of compound **1n**.

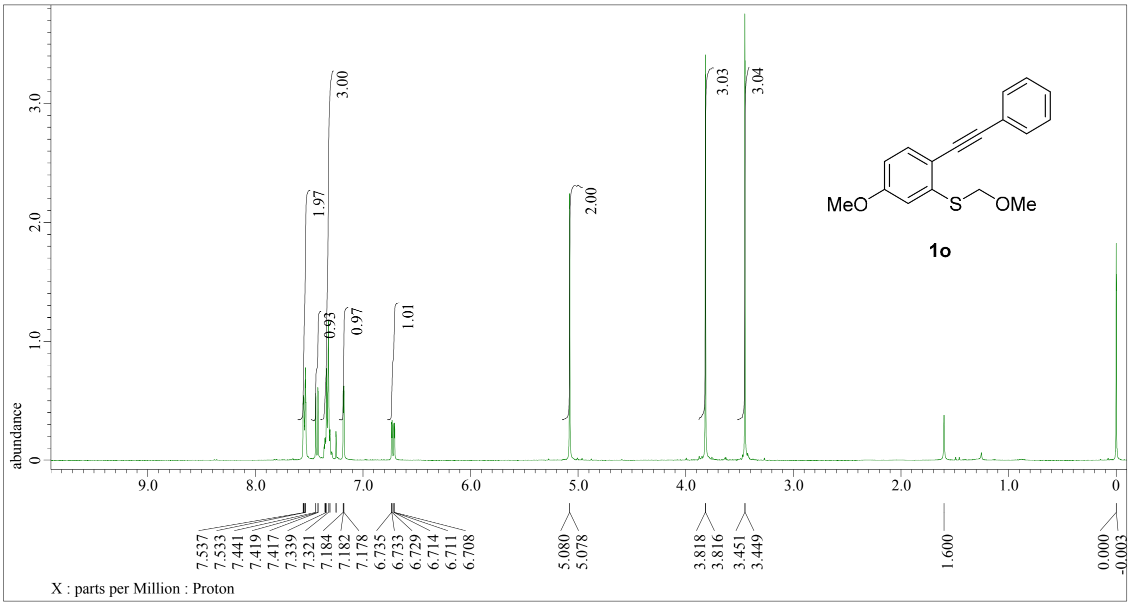

Figure S29. ^{1}H-NMR of compound **1o**.

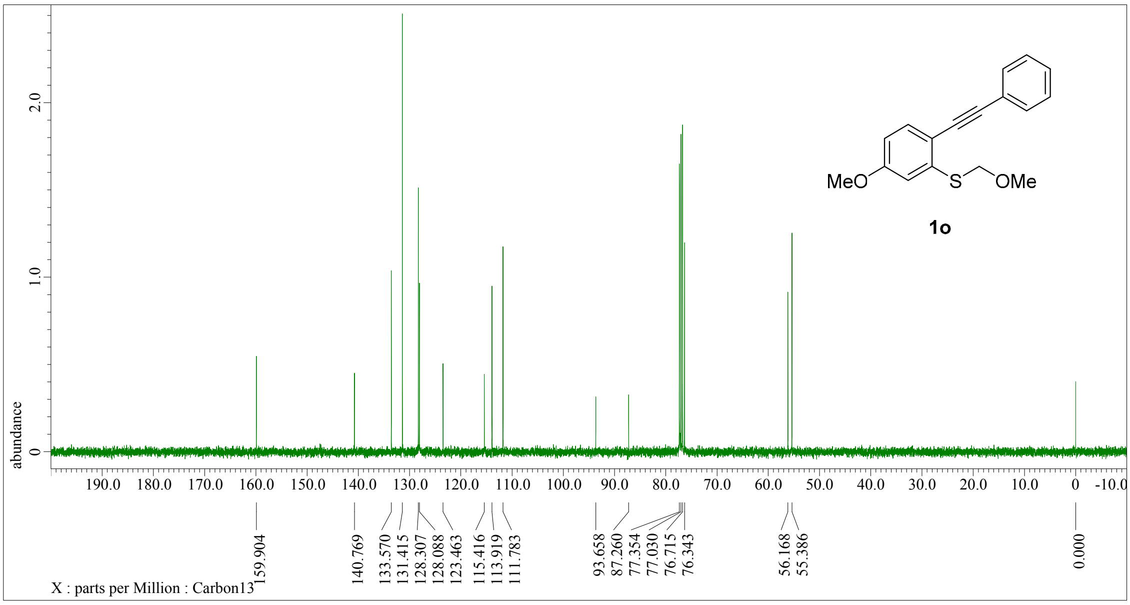

Figure S30. ^{13}C-NMR of compound **1o**.

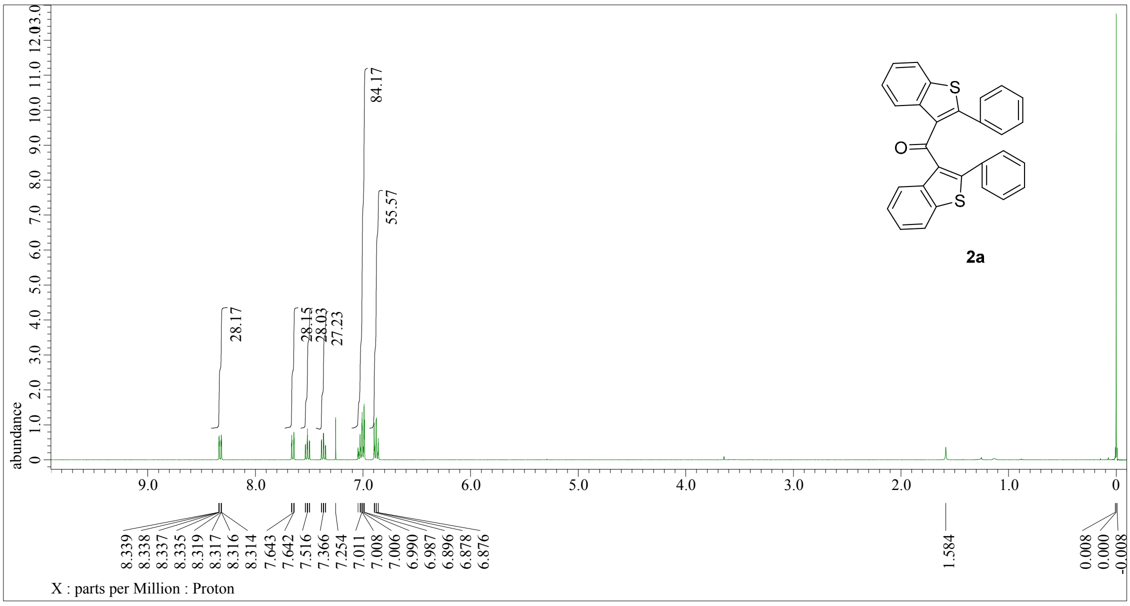

Figure S31. ^{1}H-NMR of compound **2a**.

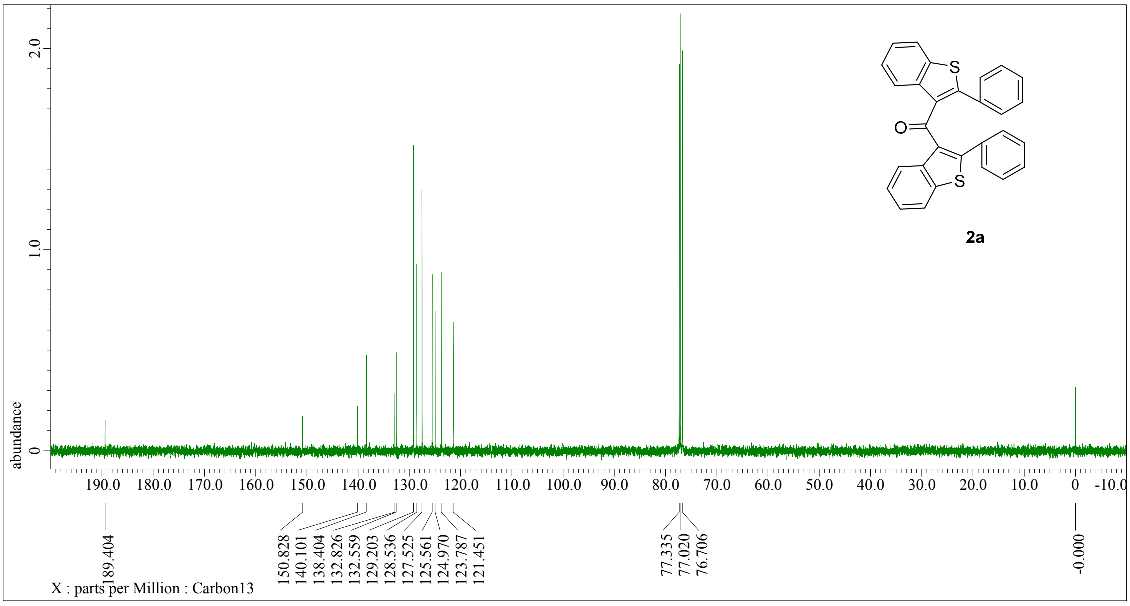

Figure S32. ^{13}C-NMR of compound **2a**.

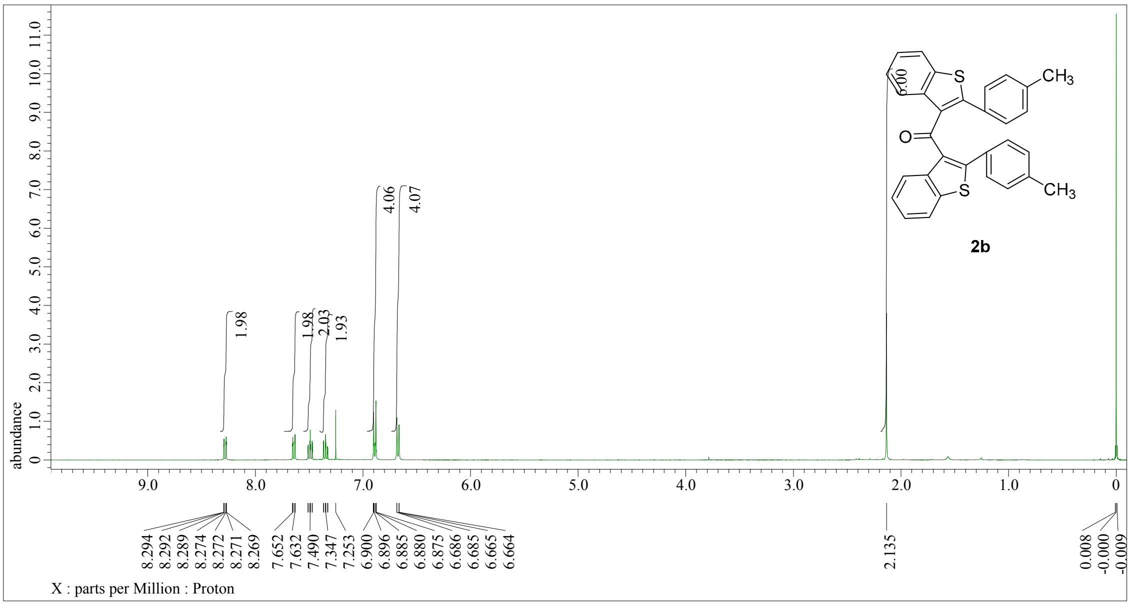

Figure S33. ^{1}H-NMR of compound **2b**.

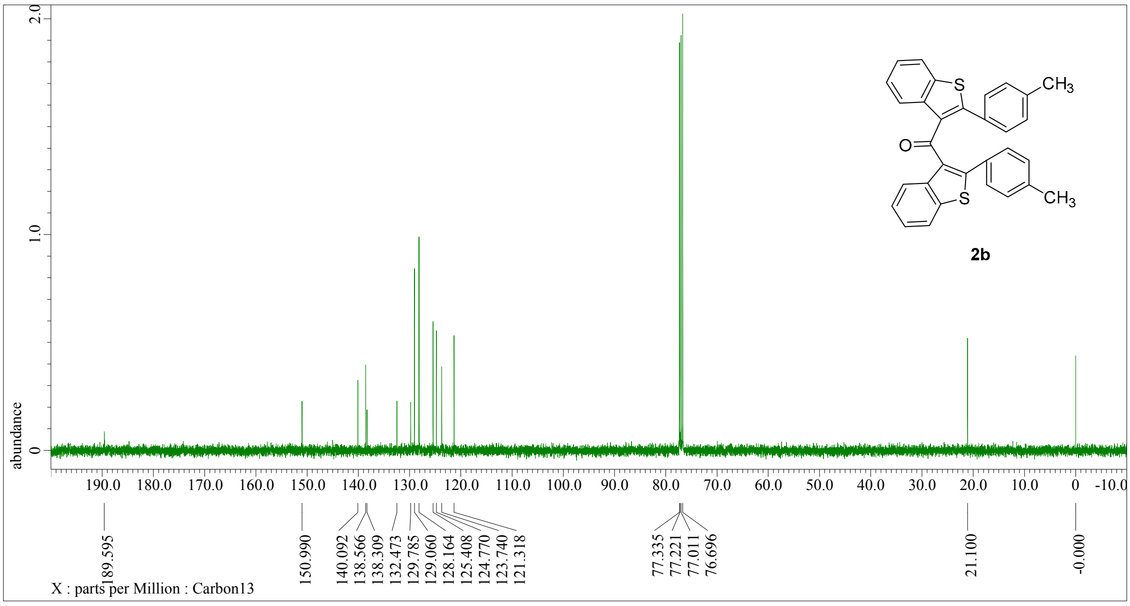

Figure S34. ^{13}C-NMR of compound **2b**.

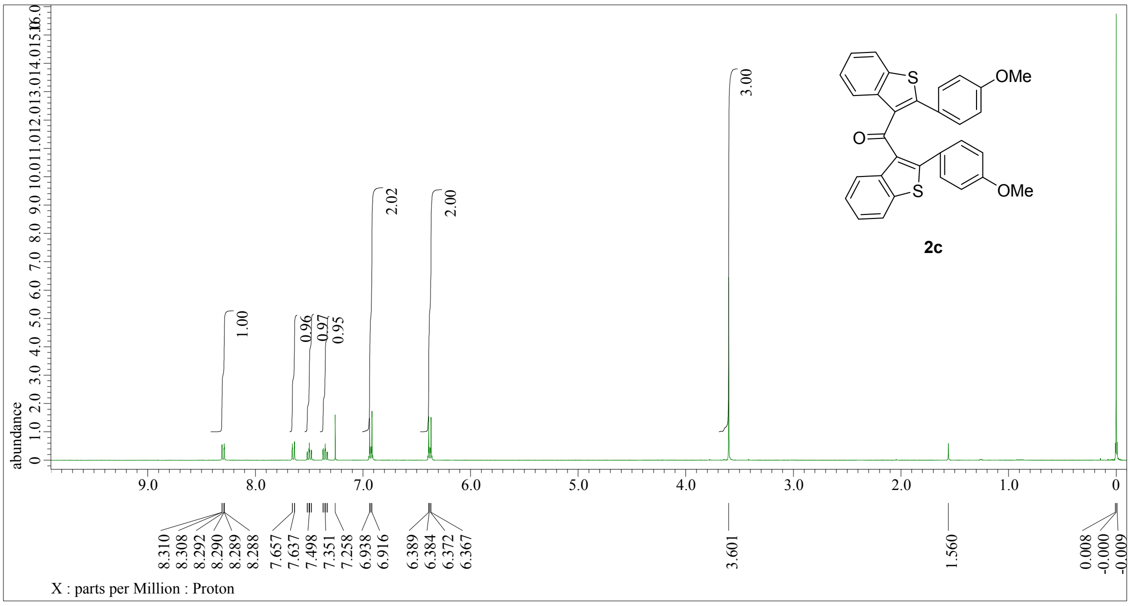

Figure S35. ^{1}H-NMR of compound **2c**.

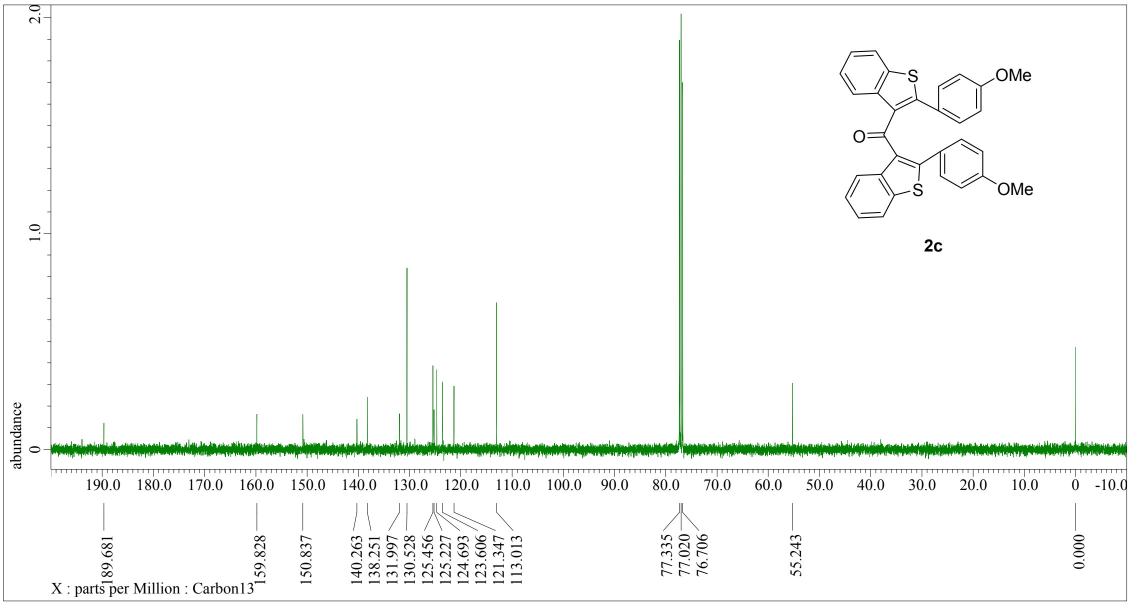

Figure S36. ^{13}C-NMR of compound **2c**.

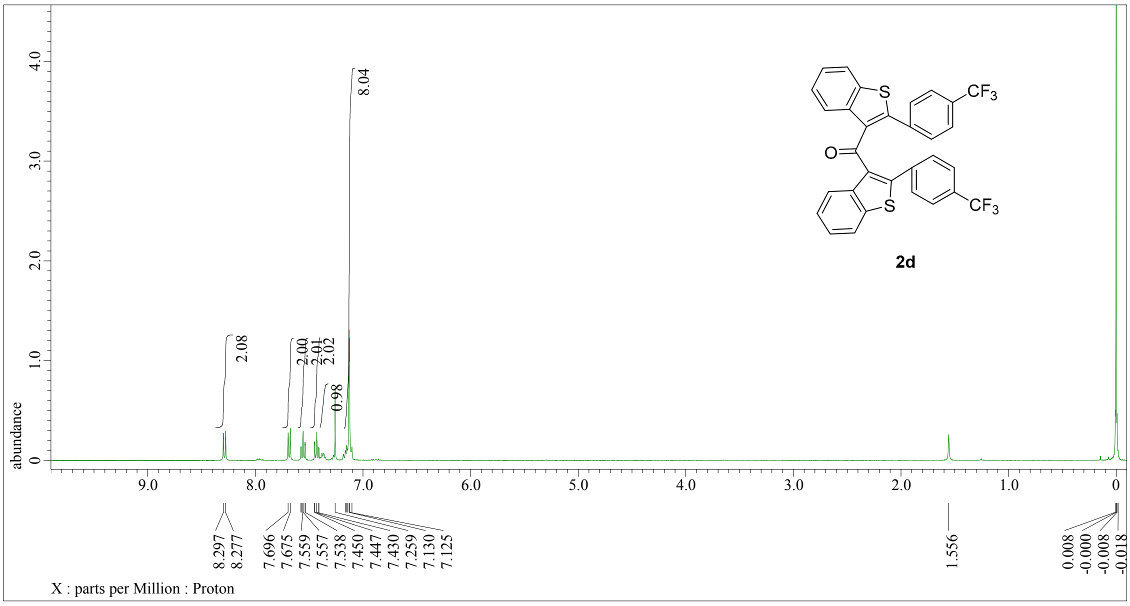

Figure S37. ^{1}H-NMR of compound **2d**.

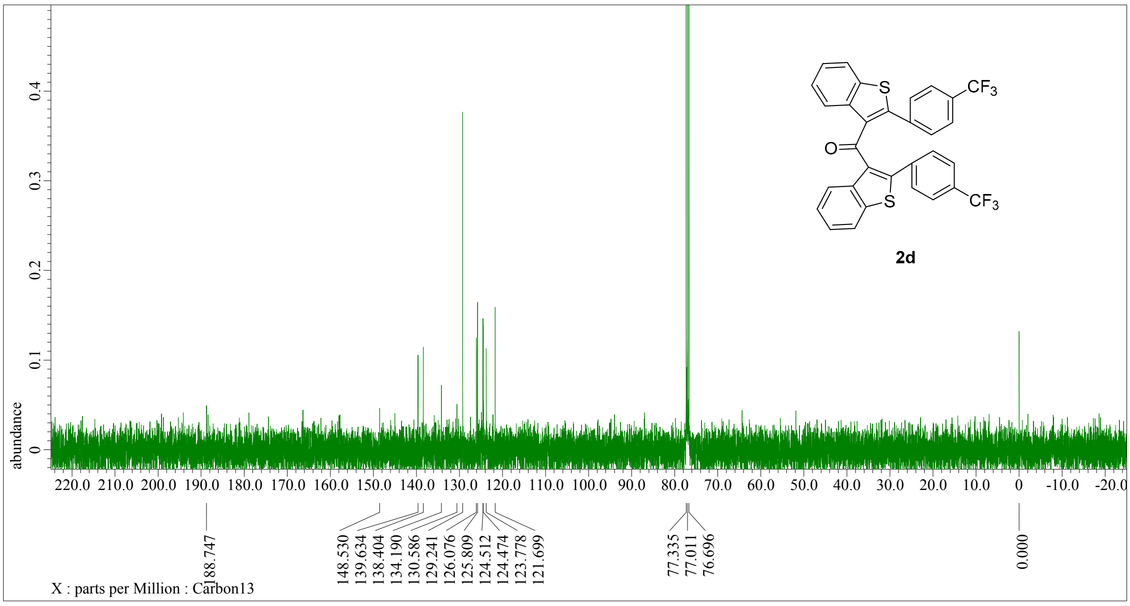

Figure S38. ^{13}C-NMR of compound **2d**.

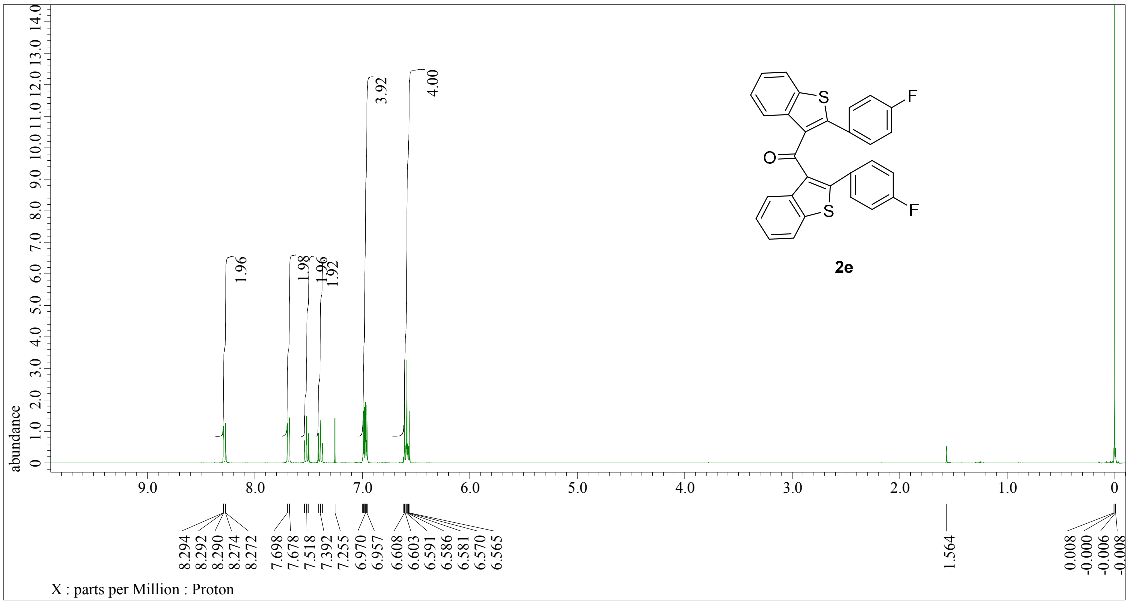

Figure S39. ¹H-NMR of compound **2e**.

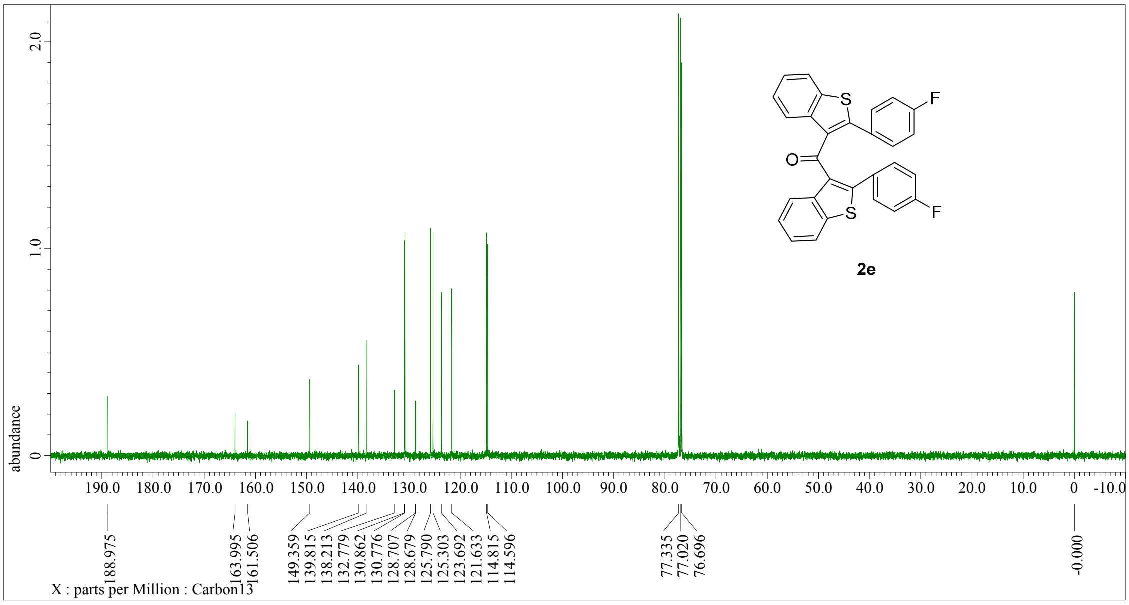

Figure S40. ¹³C-NMR of compound **2e**.

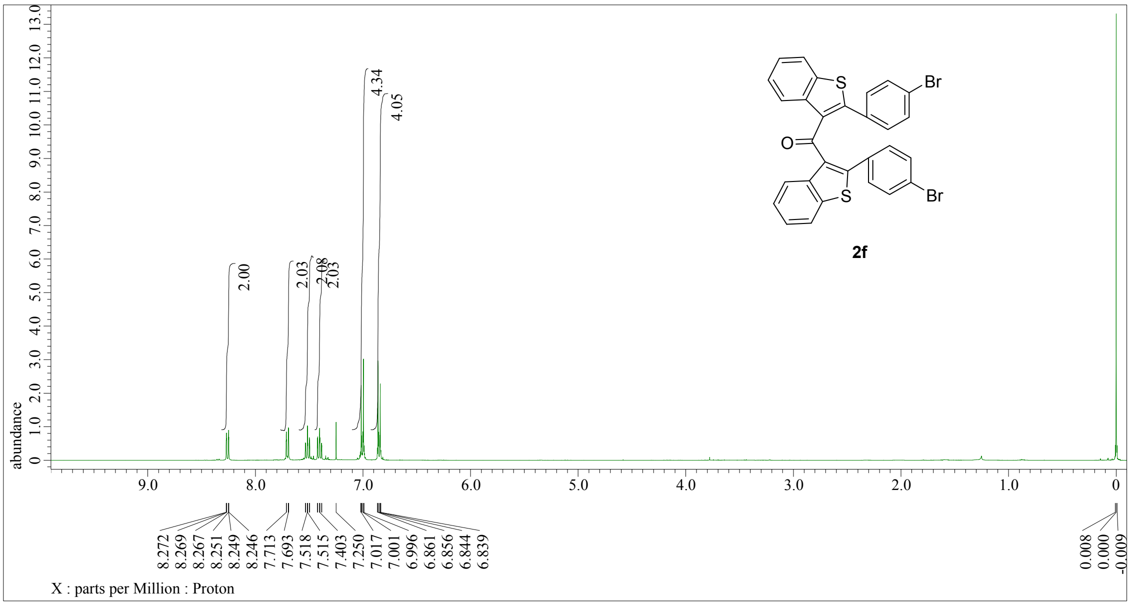

Figure S41. ^{1}H-NMR of compound **2f**.

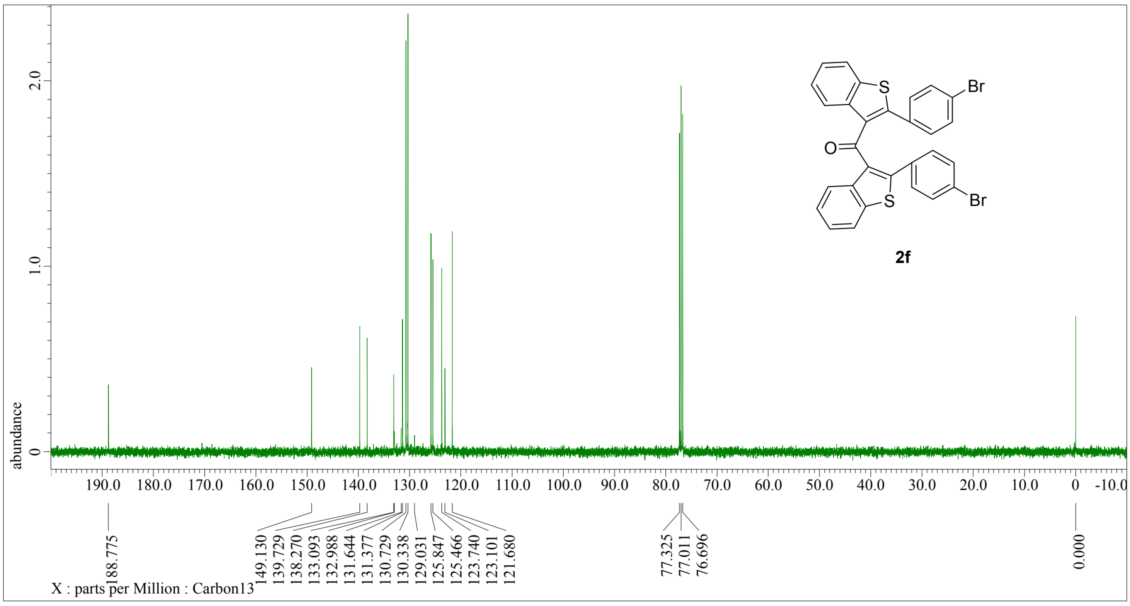

Figure S42. ^{13}C-NMR of compound **2f**.

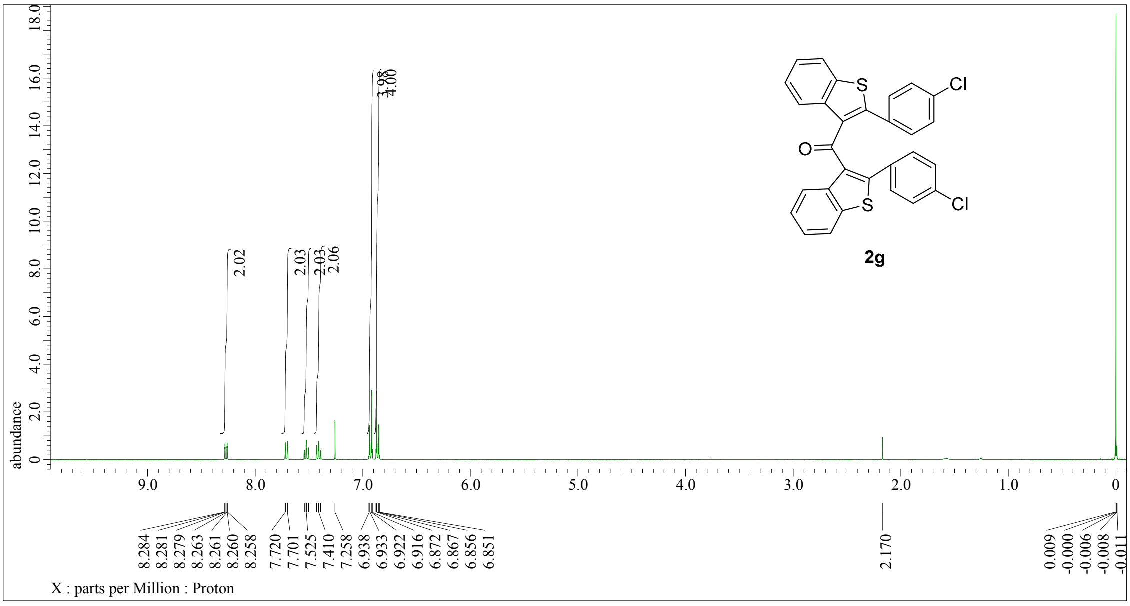

Figure S43. ^{1}H-NMR of compound **2g**.

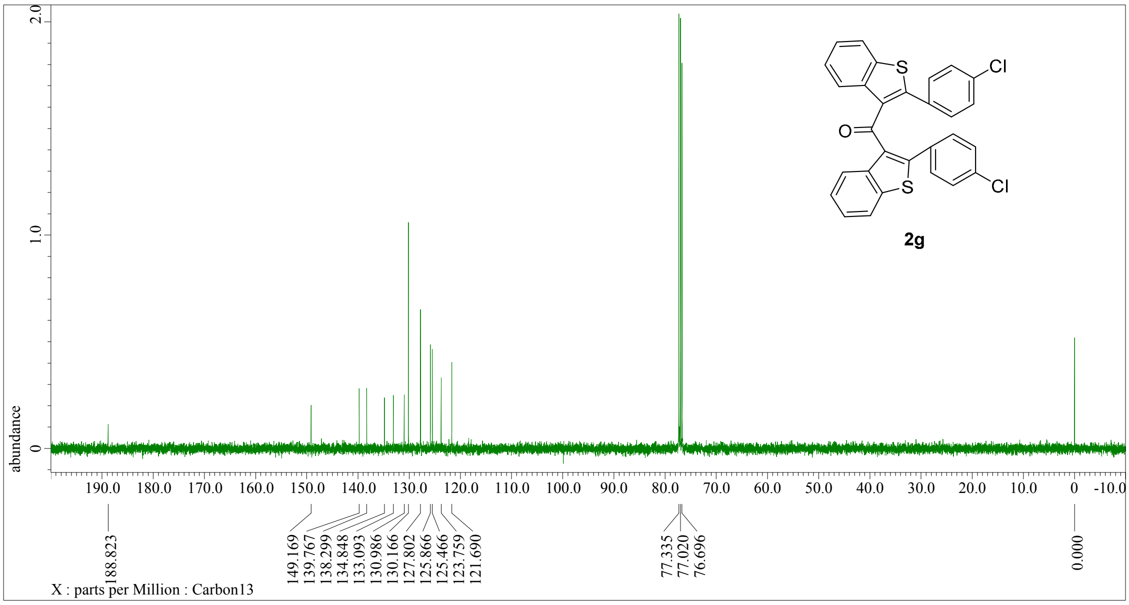

Figure S44. ^{13}C-NMR of compound **2g**.

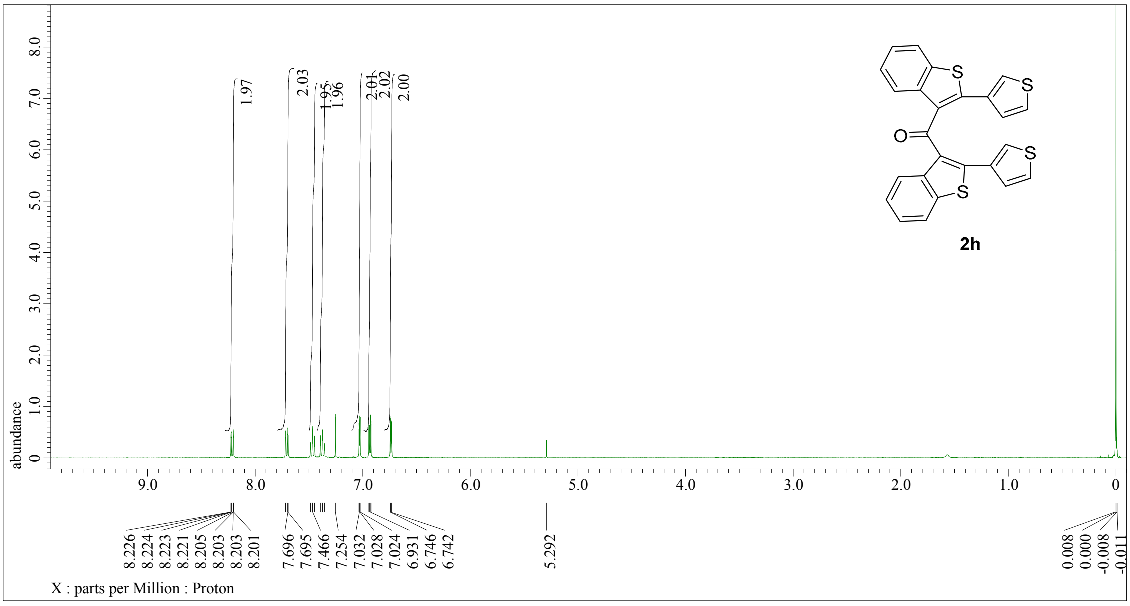

Figure S45. ^{1}H-NMR of compound **2h**.

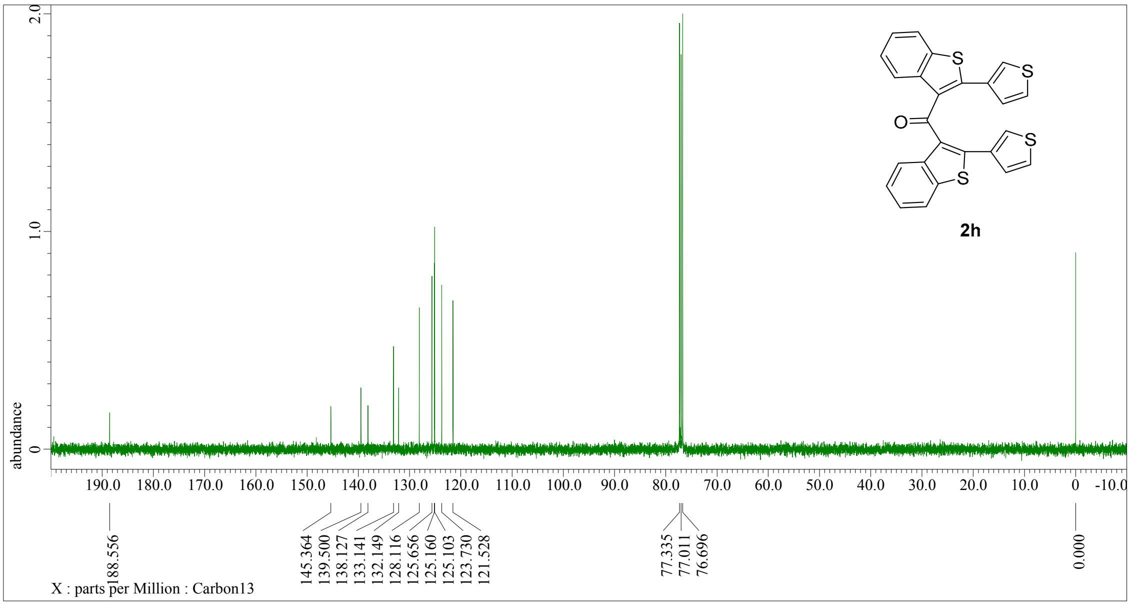

Figure S46. ^{13}C-NMR of compound **2h**.

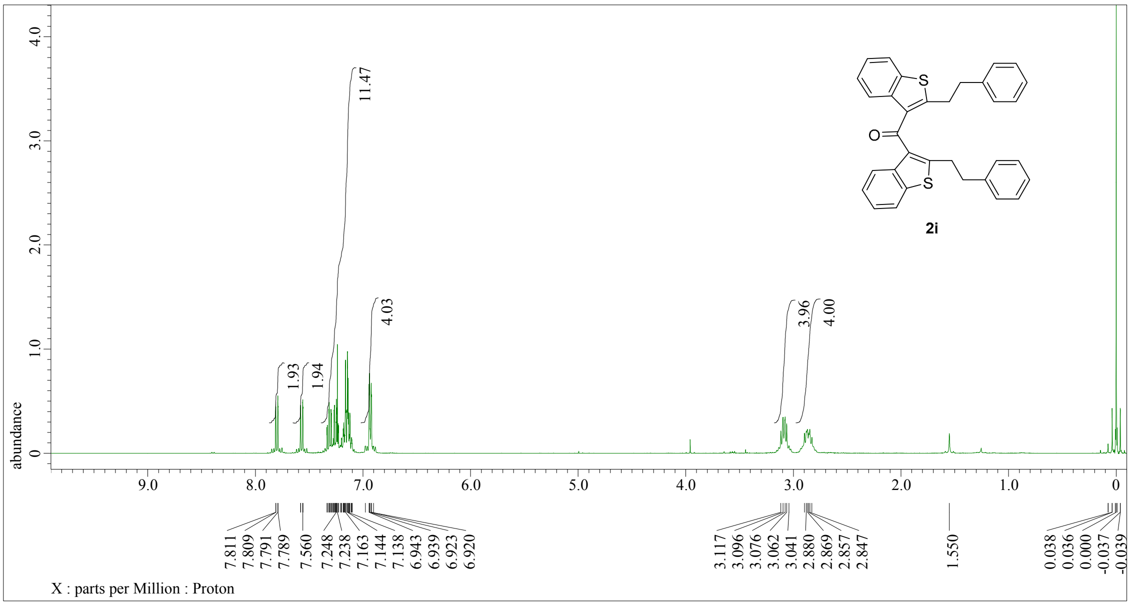

Figure S47. ^{1}H-NMR of compound **2i**.

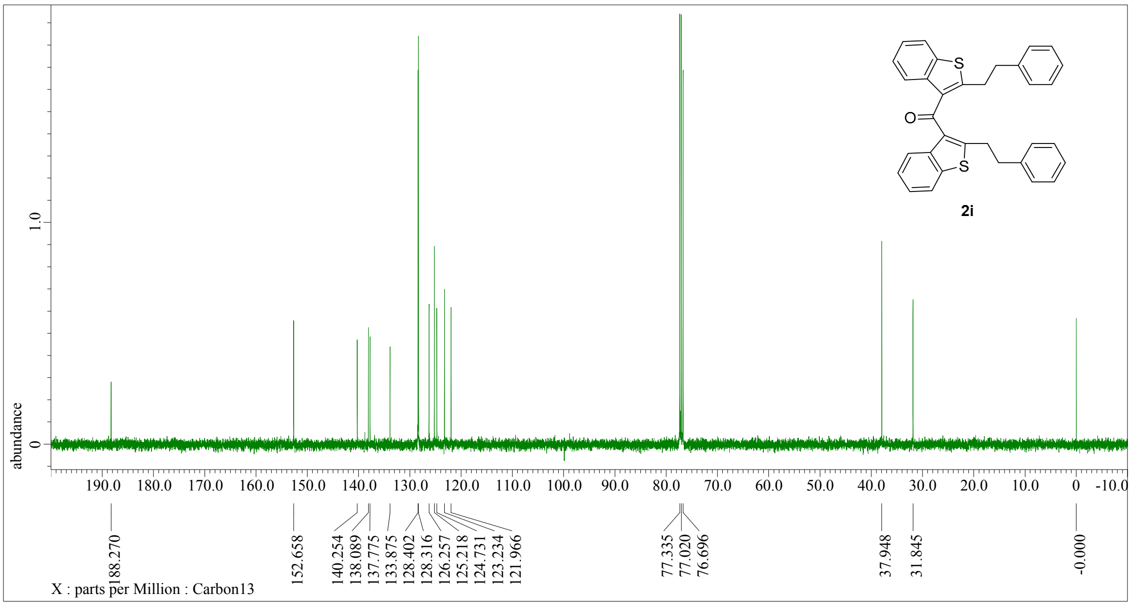

Figure S48. ^{13}C-NMR of compound **2i**.

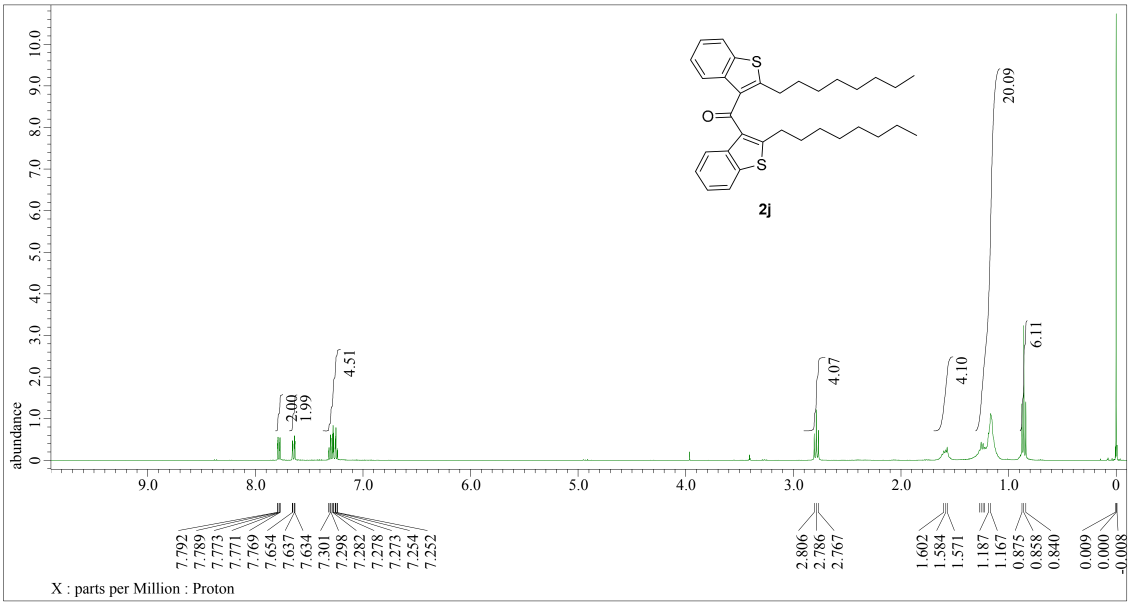

Figure S49. ^{1}H-NMR of compound **2j**.

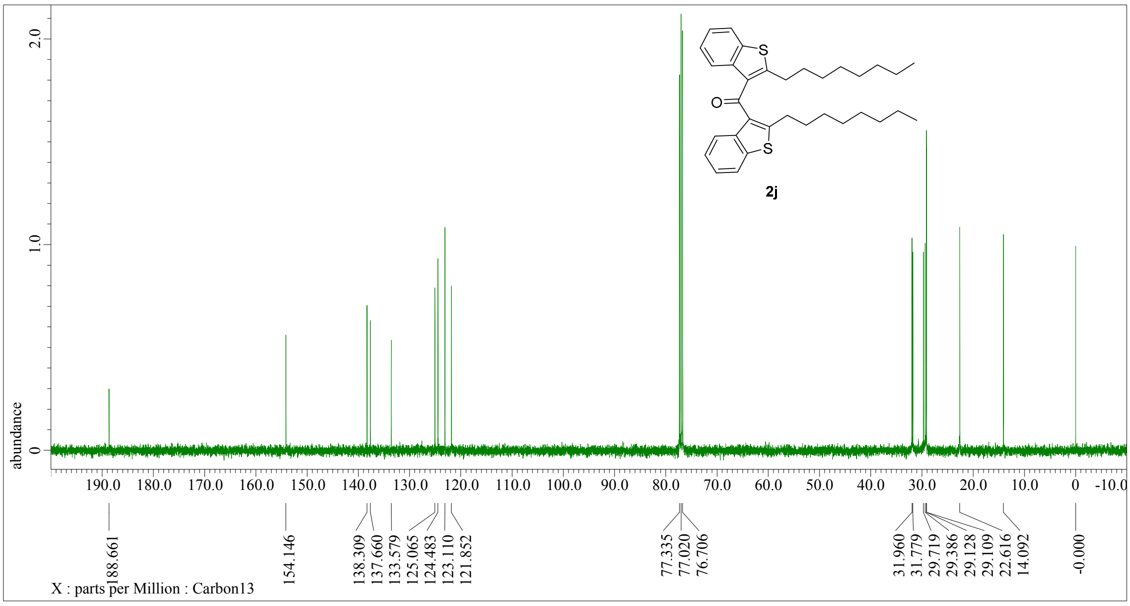

Figure S50. ^{13}C-NMR of compound **2j**.

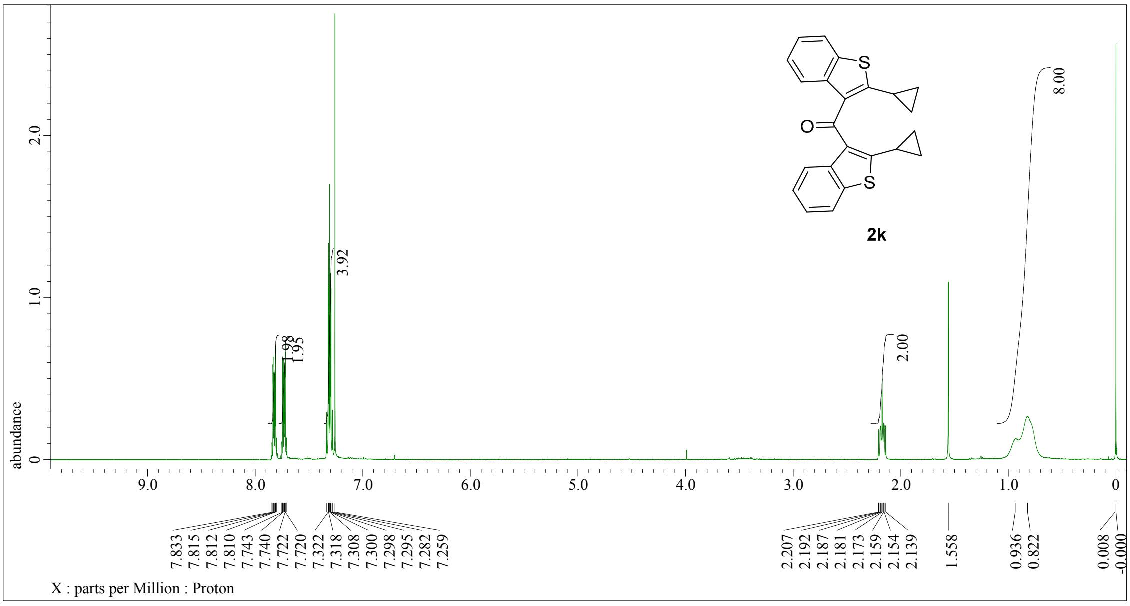

Figure S51. ^{1}H-NMR of compound **2k**.

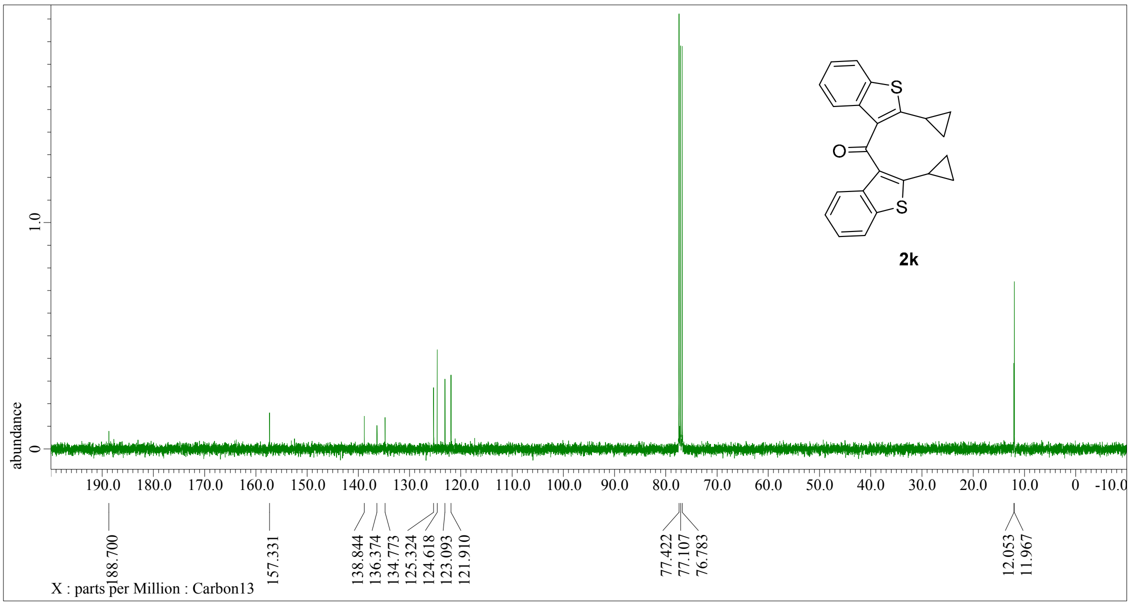

Figure S52. ^{13}C-NMR of compound **2j**.

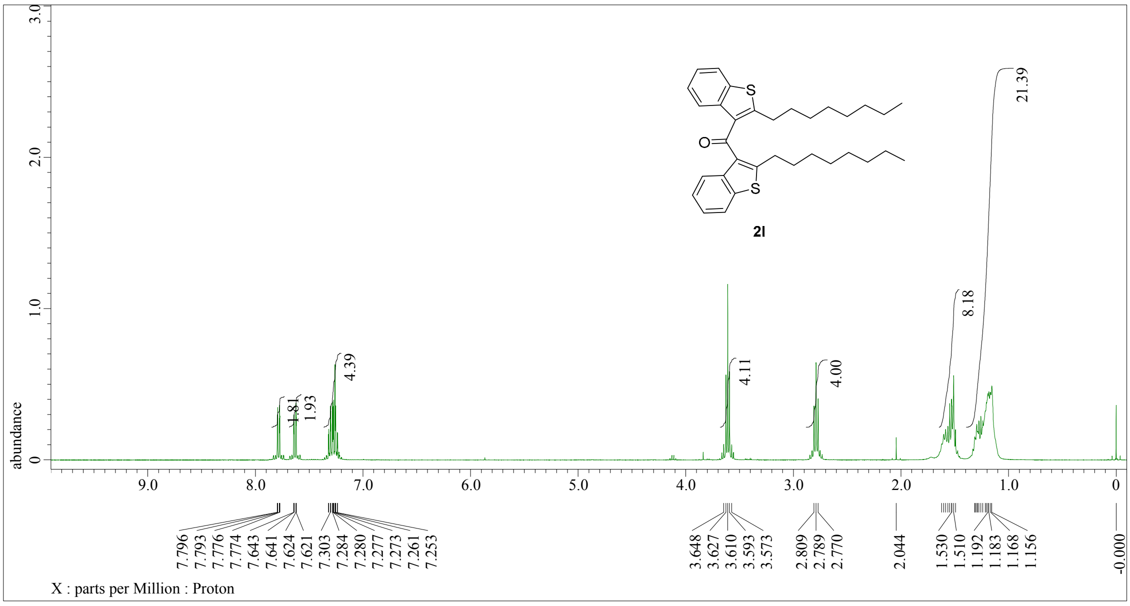

Figure S53. ^{1}H-NMR of compound **2l**.

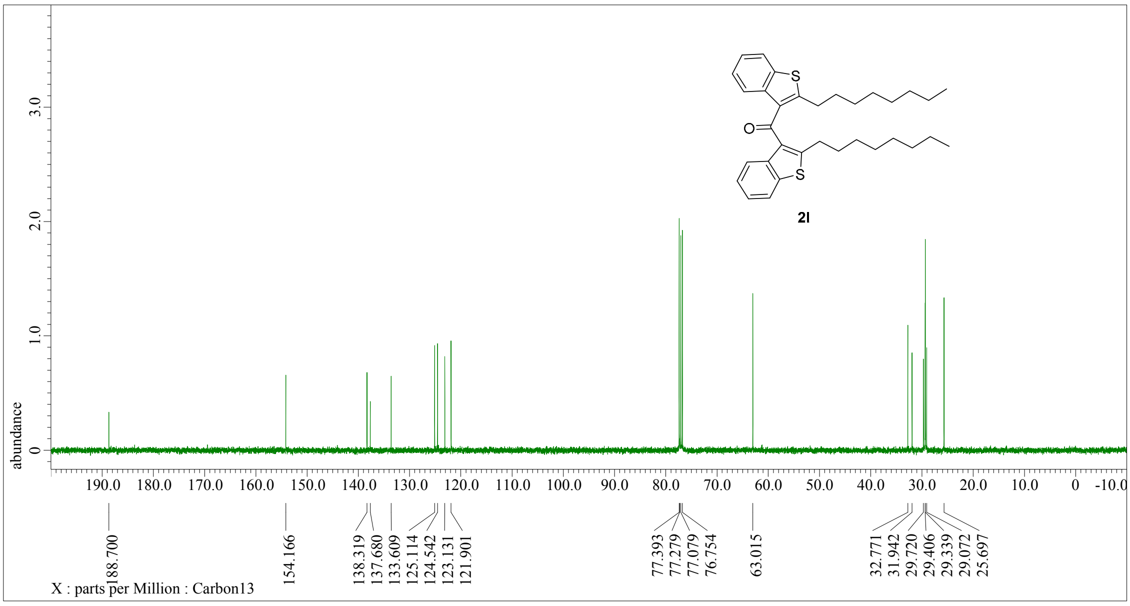

Figure S54. ^{13}C-NMR of compound **2l**.

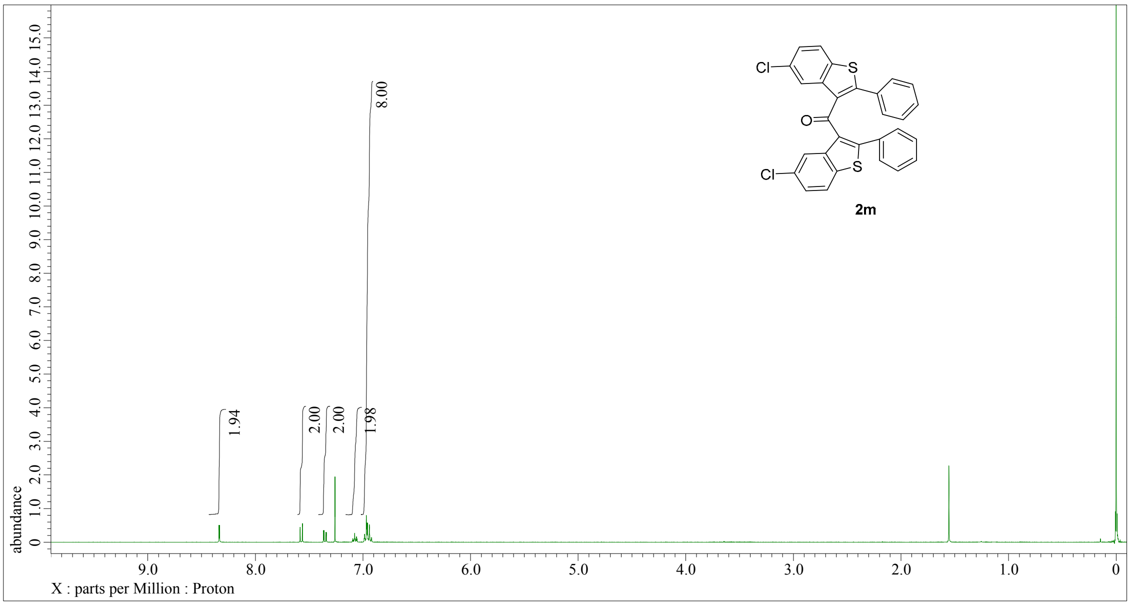

Figure S55. ^{1}H-NMR of compound **2m**.

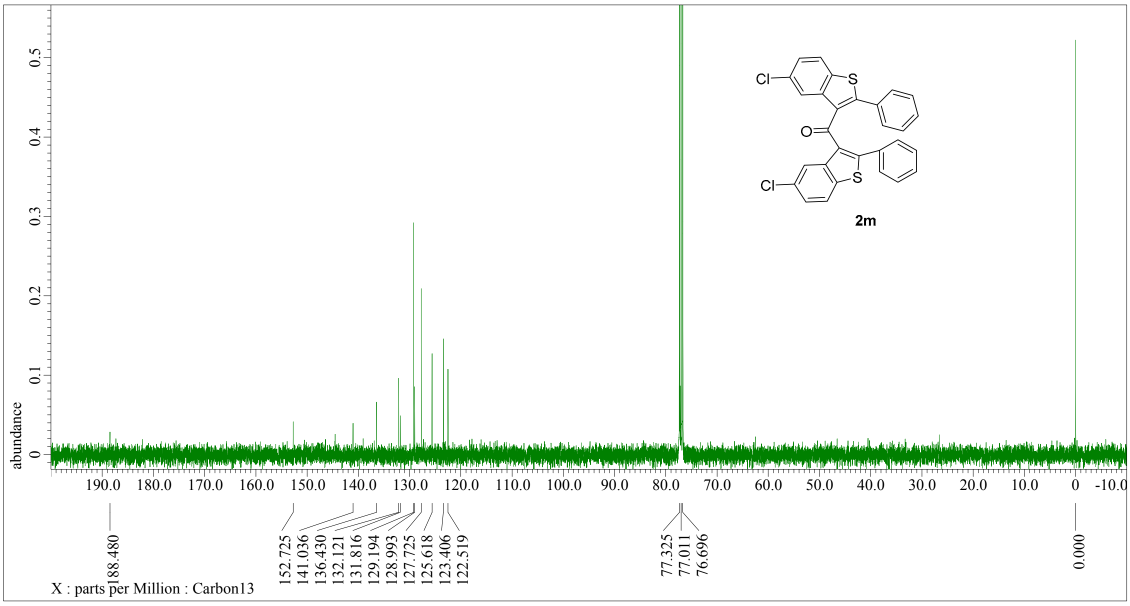

Figure S56. ^{13}C-NMR of compound **2m**.

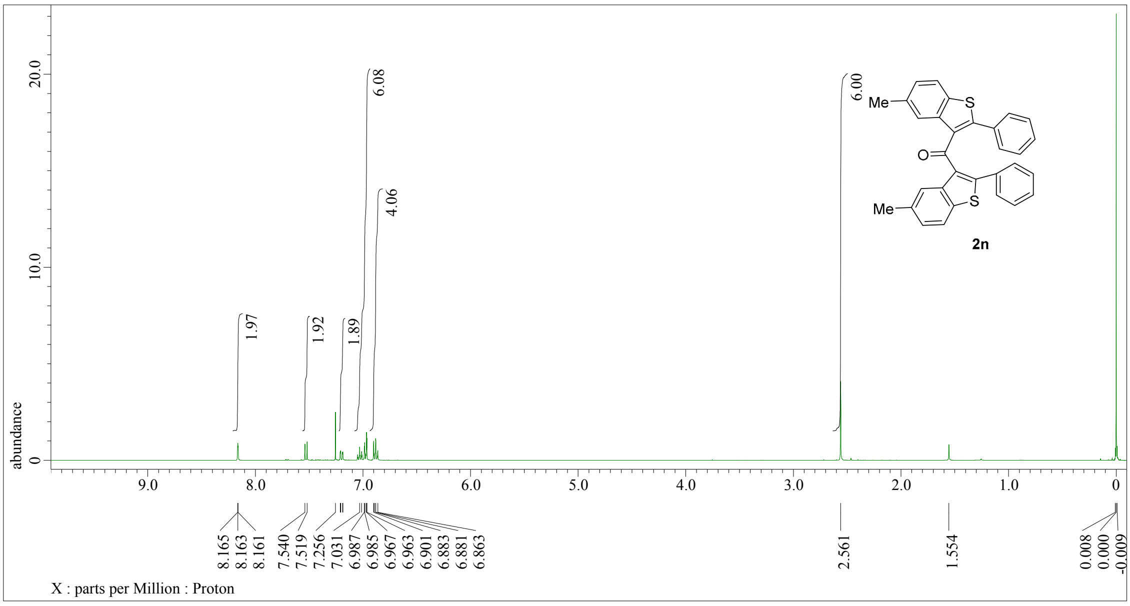

Figure S57. ^{1}H-NMR of compd **2n**.

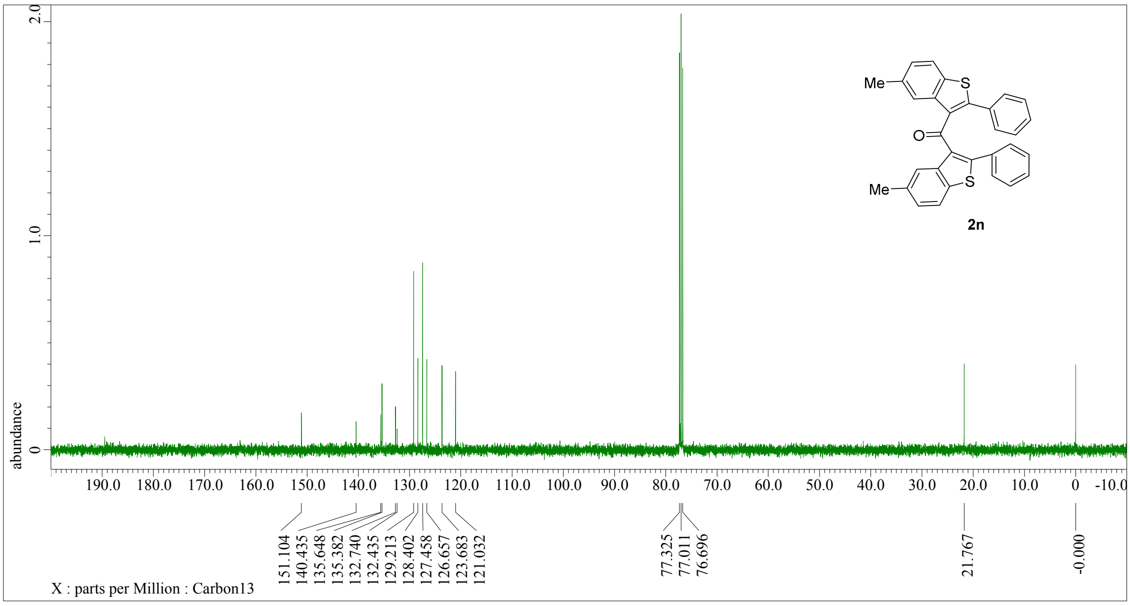

Figure S58. ^{13}C-NMR of compound **2n**.

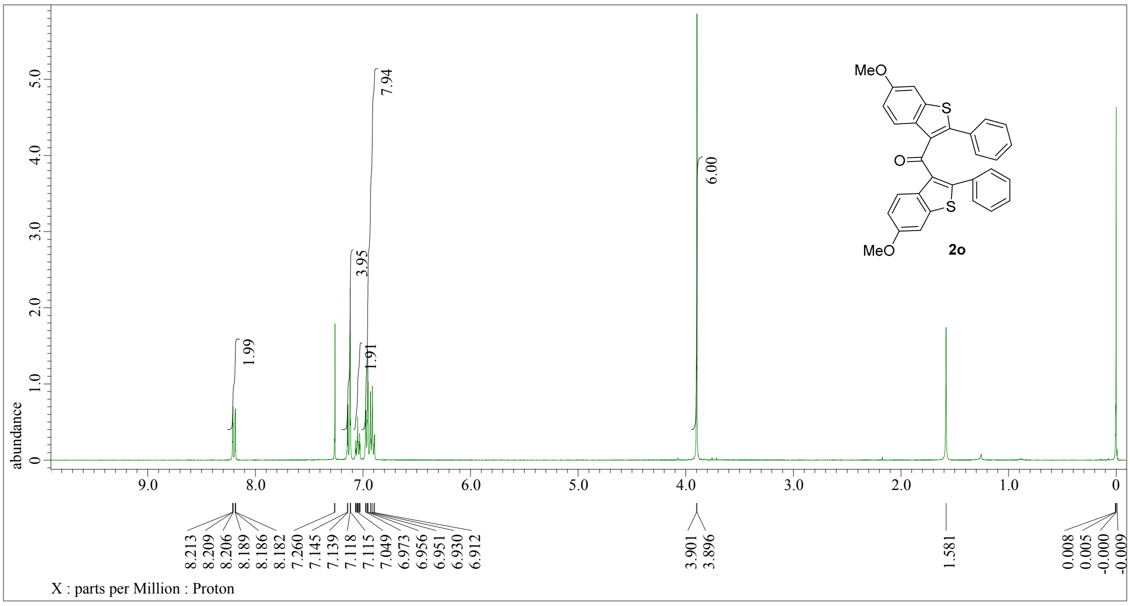

Figure S59. ^{1}H-NMR of compd **2o**.

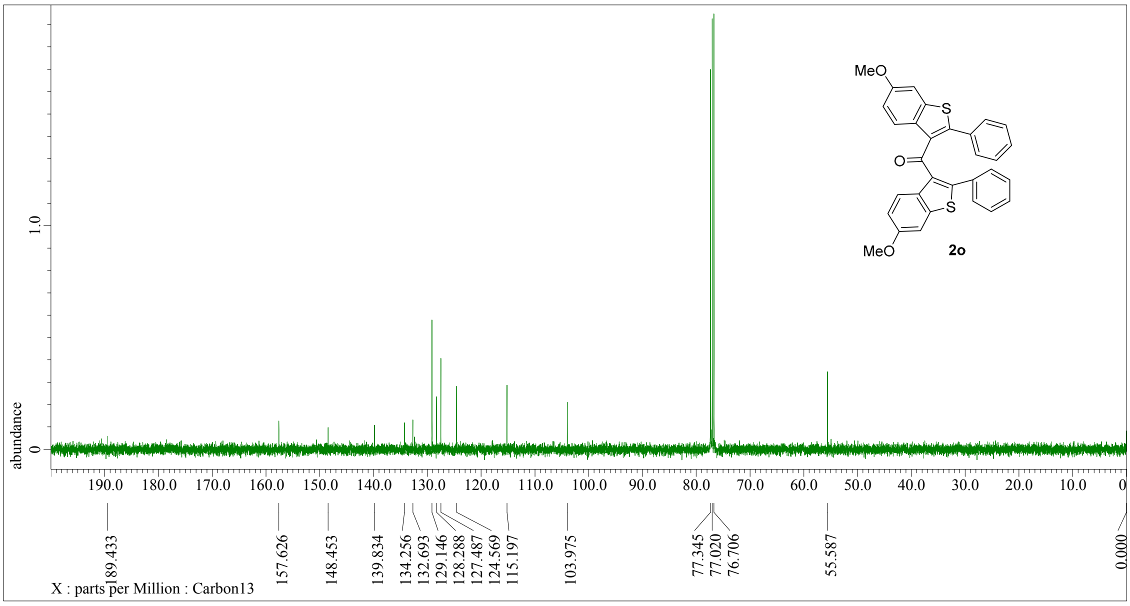

Figure S60. ^{13}C-NMR of compound **2o**.

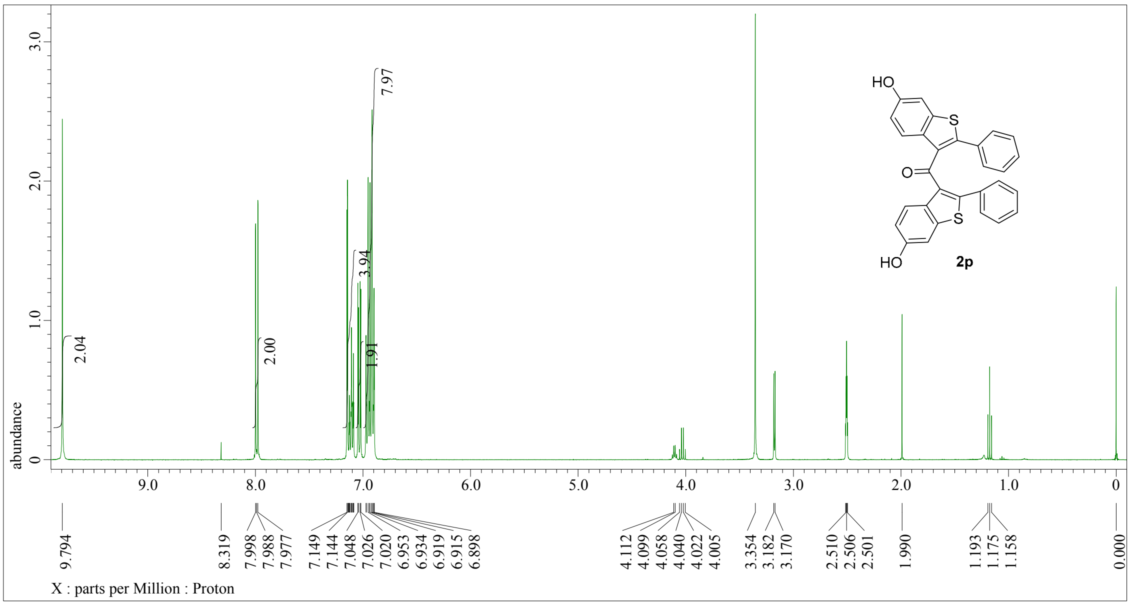

Figure S61. ^{1}H-NMR of compound **2p**.

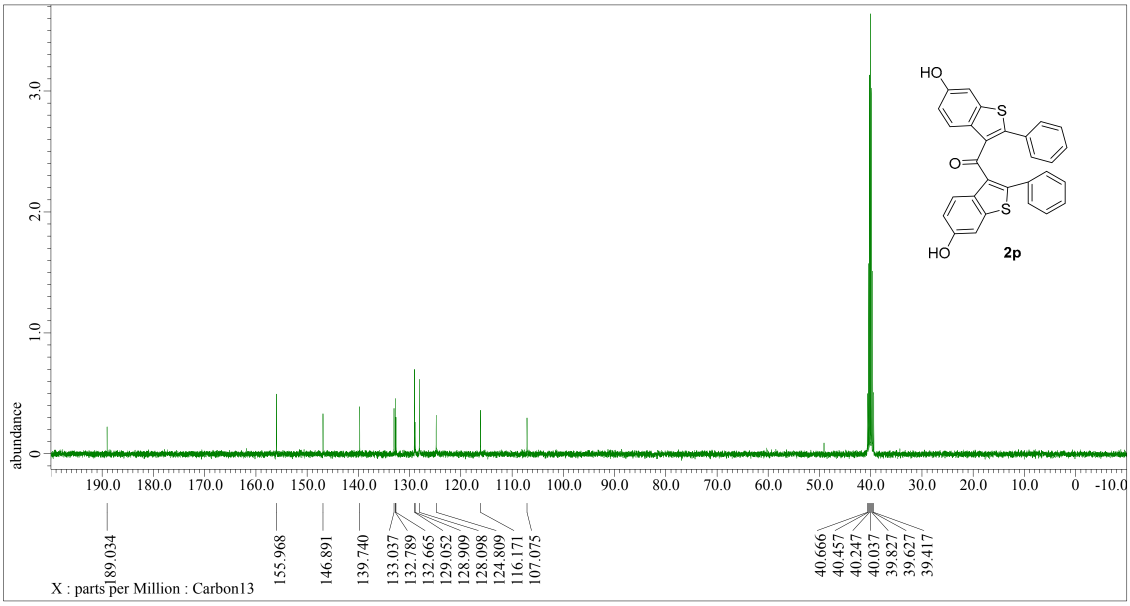

Figure S62. ^{13}C-NMR of compound **2p**.
